# A *Phytophthora infestans* RXLR effector targets plant PP1c isoforms that promote late blight disease

**DOI:** 10.1038/ncomms10311

**Published:** 2016-01-29

**Authors:** Petra C. Boevink, Xiaodan Wang, Hazel McLellan, Qin He, Shaista Naqvi, Miles R. Armstrong, Wei Zhang, Ingo Hein, Eleanor M. Gilroy, Zhendong Tian, Paul R. J. Birch

**Affiliations:** 1 Department of Cell and Molecular Sciences, James Hutton Institute, Errol Road, Invergowrie, Dundee DD2 5DA, UK; 2 Division of Plant Sciences, College of Life Science, University of Dundee (at JHI), Errol Road, Invergowrie, Dundee DD2 5DA, UK; 3 Virus-free Seedling Research Institute of Heilongjiang Academy of Agricultural Sciences, 368 Xuefu Road, Harbin 150086, China; 4 Key Laboratory of Horticultural Plant Biology (at HAU), Ministry of Education, National Center for Vegetable Improvement (Central China), Huazhong Agricultural University, Wuhan, Hubei 430070, China

## Abstract

Plant pathogens deliver effectors to alter host processes. Knowledge of how effectors target and manipulate host proteins is critical to understand crop disease. Here, we show that *in planta* expression of the RXLR effector Pi04314 enhances leaf colonization by *Phytophthora infestans* via activity in the host nucleus and attenuates induction of jasmonic and salicylic acid-responsive genes. Pi04314 interacts with three host protein phosphatase 1 catalytic (PP1c) isoforms, causing their re-localization from the nucleolus to the nucleoplasm. Re-localization of PP1c-1 also occurs during infection and is dependent on an R/KVxF motif in the effector. Silencing the *PP1c* isoforms or overexpression of a phosphatase-dead PP1c-1 mutant attenuates infection, demonstrating that host PP1c activity is required for disease. Moreover, expression of PP1c–1mut abolishes enhanced leaf colonization mediated by *in planta* Pi04314 expression. We argue that PP1c isoforms are susceptibility factors forming holoenzymes with Pi04314 to promote late blight disease.

## Abstract (web summary)

*Phytophtora infestans* is the causative agent of late blight disease in plants. Here Boevink *et al*. provide evidence that host protein phosphatases are targeted by a *P. infestans* effector protein and are required for disease development.

## Graphical abstract

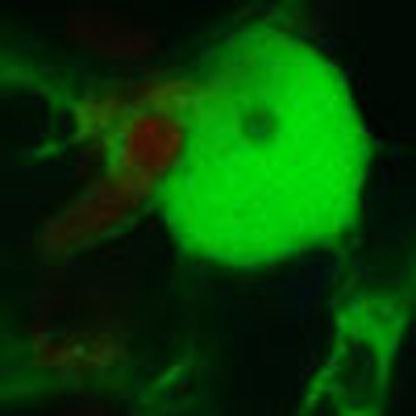

Detection of conserved microbial molecules (microbe-associated molecular patterns) or the products generated by microbial degradation of host cell walls (damage-associated molecular patters) at the plasma membrane by plant pattern recognition receptors, leads to pattern-triggered immunity (PTI)^1^. Host-adapted pathogens have evolved to manipulate processes in plants that result in suppression of PTI^2^. Among the strategies employed by pathogens to suppress PTI is the secretion of effectors that act either in the apoplast or are delivered inside living plant cells to target regulatory host proteins. Our understanding of how effectors manipulate plant targets has been led by studies of proteins translocated into plant cells by the bacterial type III secretion system^1^,^3^,^4^. Studies to identify and characterize effector targets, while in their infancy, are revealing fascinating insights into host processes that are targeted, and into the mechanisms of effector activity^5^. Relatively little is understood about the targets of effectors from filamentous pathogens such as fungi and oomycetes, and yet these microorganisms are arguably the major causes of plant disease, posing a serious threat to food security.

Oomycetes, including plant pathogens of considerable economic and environmental impact, display a range of modes of infection, from obligate biotrophy to necrotrophy. They deliver a variety of apoplastic and intracellular (cytoplasmic) effectors during infection^6^. The best studied cytoplasmic oomycete effectors are the RXLR class, which contain a conserved Arg-x-Leu-Arg (RXLR) motif required for their translocation from the pathogen to the inside of host plant cells^7^,^8^. Following the discovery of RXLR effectors, an essential ‘next step' involves their use as probes to reveal the host proteins, processes and mechanisms that are targeted to promote susceptibility.

Plant immunity involves a complex network of inter-linked signalling and regulatory processes. Regulation occurs at every level, from differential transcript accumulation and processing, through to protein modification and turnover. Important post-translational modifications (PTMs) include ubiquitylation and phosphorylation. Ubiquitin E3 ligases play roles in both negative and positive regulation of PTI^9^. Kinases can act to positively regulate PTI at several levels, from the receptor complex^10^, to signal transduction^11^ and the activation of transcriptional regulators^12^, through to the complex cross-talk between phytohormone signalling pathways^13^. Conversely, phosphatase PP2A has recently been shown to act as a negative regulator of plant immunity^14^. Indeed, a bacterial type III effector, HopAO1 from *Pseudomonas syringae*, is a tyrosine phosphatase that inactivates the pathogen-associated molecular pattern (PAMP) receptor elongation factor Tu receptor (EFR)^15^, demonstrating that dephosphorylation is a potential strategy to establish susceptibility.

In recent years, oomycete RXLR effectors have been shown to either target regulatory host proteins involved in PTM, or to alter PTMs associated with immunity. AVR3a from the potato blight pathogen *Phytophthora infestans*, targets the ubiquitin E3 ligase, CMPG1, a positive regulator of cell death following perception of a range of pathogen elicitors^16^,^17^. The *Hyaloperonospora arabidopsidis* effector HaRxL44 attenuates salicylic acid (SA)-triggered immunity by targeting a subunit of the mediator complex, Med19a, for proteasomal degradation, although how this is achieved is unknown^18^. More recently, three RXLR effectors from *P. infestans* have been shown to suppress PTI mediated by the bacterial PAMP flg22 by preventing mitogen-activated protein kinase (MAPK) phosphorylation and activation^19^, and a further *P. infestans* RXLR effector, PexRD2, interacts with the kinase domain of MAP3Kɛ to prevent signal transduction leading to cell death mediated by the receptor Cf4 (ref. 20). These examples demonstrate that oomycetes can either directly suppress PTI by altering PTMs that positively regulate immunity, or, in the case of HaRxL44, can promote ubiquitination of an immune regulator to affect its degradation.

In this study, we focus on an RXLR effector from *P. infestans*, PITG_04314/PexRD24 (Pi04314), which is predicted to contain the WY structural fold characteristic of RXLR effectors^21^, and is strongly upregulated during the biotrophic phase of infection in all isolates tested^22^,^23^,^24^, suggesting that it is a ‘core' effector. Pi04314 is recognized by resistances in a range of accessions of the non-host plant pepper^25^, a solanaceous relative of the *P. infestans* crop hosts potato and tomato, and the model host *Nicotiana benthamiana*, implicating its recognition in non-host immunity.

Here, we show that when expressed in *N. benthamiana* or potato, Pi04314 acts to enhance *P. infestans* leaf colonization. We propose a model whereby Pi04314 interacts with isoforms of host protein phosphatase type 1c (PP1c), mimicking a regulatory subunit and causing their re-localization within the host nucleus. We suggest that Pi04134 forms holoenzymes with PP1c isoforms and acts to promote late blight by attenuating jasmonic acid (JA)- and SA-mediated transcriptional responses of the host plant.

## Results

### *In planta* expression of Pi04314 enhances infection

Candidate RXLR effector PITG_04314/PexRd24 (Pi04314) is upregulated strongly and specifically during the biotrophic phase of infection in all *P. infestans* isolates tested^22^,^23^,^24^. We confirmed its upregulation, during the early stages of infection (24–72 h post inoculation) of *N. benthamiana* using quantitative reverse transcription (qRT)–PCR in isolate 88069, which we use for laboratory studies (Supplementary Fig. 1a). *N. benthamiana* is an extensively used model host for *P. infestans*, as it allows transient *Agrobacterium*-mediated gene expression, virus-induced gene silencing (VIGS) and non-invasive confocal microscopy to aid functional characterization of molecular interactions between pathogen and host^16^,^19^,^20^,^26^.

**Figure 1:**
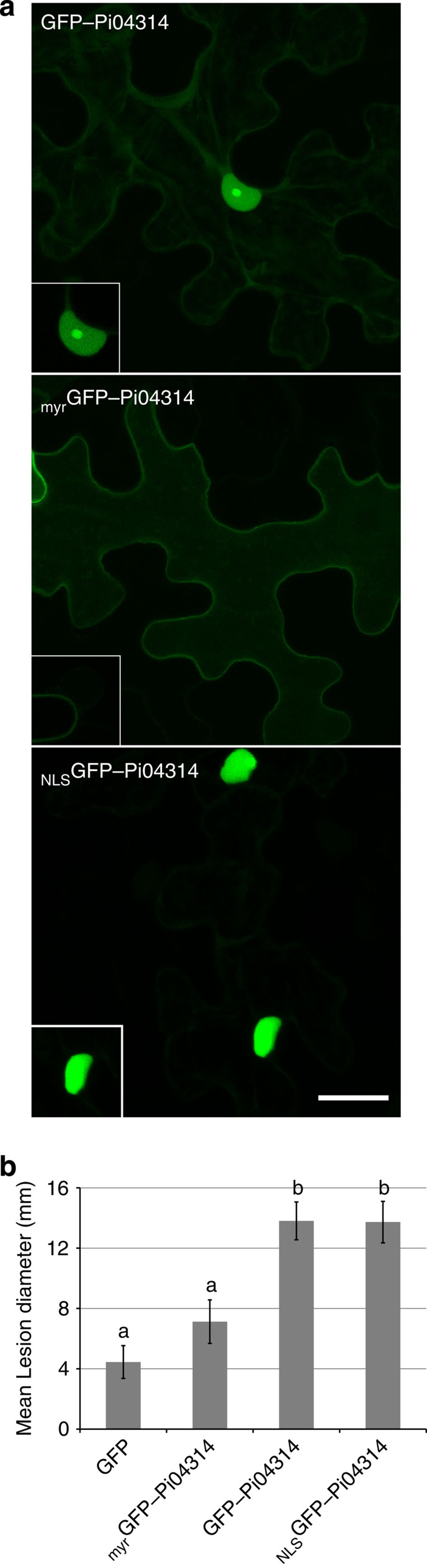
The nuclear location of effector fusion GFP–Pi04314 is important for its ability to promote infection. (**a**) Images are projections of confocal Z series of GFP–Pi04314 and the forms of the fusion with added targeting signals; _myr_GFP–Pi04314 and _NLS_GFP–Pi04314 to decrease or increase the level of the effector fusion in the nucleus, respectively. Inset images are single optical sections through the nuclei of the cells. Scale bar, 20 μm. (**b**) GFP–Pi04314 and _NLS_GFP–Pi04314 are able to promote *P. infestans* growth following *Agrobacterium*-mediated expression compared with a GFP control. Lesion diameter was not significantly different between _myr_GFP–Pi04314-expressing plants and the control. Error bars are standard error and the graph represents the combined data from three biological replicates (*n*=56 per construct). Letters on the graph denote statistically significant differences (ANOVA, *P*≤0.005).

To determine the subcellular localization of Pi04314, an N-terminal green fluorescent protein (GFP) fusion to Pi04314 was transiently expressed in *N. benthamiana*. GFP–Pi04314, which was stable *in planta* (Supplementary Fig. 1b), accumulated strongly in the nucleoplasm and the nucleolus, with additional cytoplasmic background (Fig. 1a; Supplementary Fig. 1c). To evaluate whether Pi04314 plays a role in contributing to *P. infestans* virulence, GFP–Pi04314 was transiently expressed in one-half of an *N. benthamiana* leaf with free GFP expressed in the other. The two halves of the leaves were drop-inoculated with *P. infestans* zoospores, and lesion size was measured at 7 days post inoculation (d.p.i.). The halves of leaves expressing GFP–Pi04314 were found to promote significantly (analysis of variance (ANOVA), *P*<0.001) larger *P. infestans* lesions compared with free GFP, suggesting that Pi04314 activity inside host cells is beneficial to infection (Fig. 1b; Supplementary Fig. 2).

To further investigate the site of Pi04314 activity within host cells, the effector was expressed with an N-terminal myristoylation signal (_myr_GFP–04314), as described previously^19^, to associate it with plant membranes, thus directing it away from the nucleus. This strategy was selected in preference of adding a nuclear export signal as, with a nuclear export signal, the effector would still enter the nucleus before being exported^27^. The _myr_GFP–04314 fusion was indeed largely exempted from the nucleus (Fig. 1a; Supplementary Fig. 1c). In contrast, to focus Pi04314 in the nucleus an N-terminal nuclear localization signal was added (_NLS_GFP–Pi04314)^28^. The _NLS_GFP–Pi04314 fusion protein was detected primarily in the nucleus, and remained stable *in planta* (Fig. 1a; Supplementary Fig. 1c). _myr_GFP–Pi04314 and _NLS_GFP–Pi04314 fusions were expressed transiently in *N. benthamiana* and the leaves were challenged with *P. infestans*. Figure 1b shows that expression of _myr_GFP–Pi04314 did not cause a statistically significant increase in infection compared with the free GFP control (ANOVA, *P*=0.406), whereas GFP–Pi04314 and _NLS_GFP–Pi04314 support comparable *P. infestans* lesion sizes (ANOVA, *P*=0.964), which are higher than either the free GFP control (ANOVA, *P*<0.001) or than _myr_GFP–Pi04314 (ANOVA, *P*=0.006 and *P*=0.007, respectively). On the basis of these observations, we suggest that localization of Pi04314 in the host nucleus is required, and sufficient, to promote enhanced leaf colonization by *P. infestans*.

### Pi04314 interacts with three isoforms of PP1c

To identify possible host targets of Pi04314 a yeast-2-hybrid (Y2H) library created from infected potato cDNA^16^ was screened to a depth of 2.6 million yeast co-transformants with a GAL4 DNA-binding domain-Pi04314 fusion construct (‘bait'). Eighteen yeast colonies were recovered from selection plates that contained GAL4 activation domain (‘prey') fusions, sequences from which correspond to three distinct isoforms of potato PP1c family proteins, hereafter called StPP1c-1, StPP1c-2 and StPP1c-3. A phylogenetic tree based on a trimmed alignment of the potato proteins with *Arabidopsis* type-one protein phosphatases (TOPPs) indicated that StPP1c-1, StPP1c-2 and StPP1c-3 cluster with AtTOPP1, AtTOPP2, AtTOPP4 and AtTOPP5 (Supplementary Fig. 3). Expression levels of these three *StPP1c* genes are not significantly altered during the first 2 days of *P. infestans* infection (Supplementary Fig. 3), providing no indication that they are immune-responsive.

**Figure 2:**
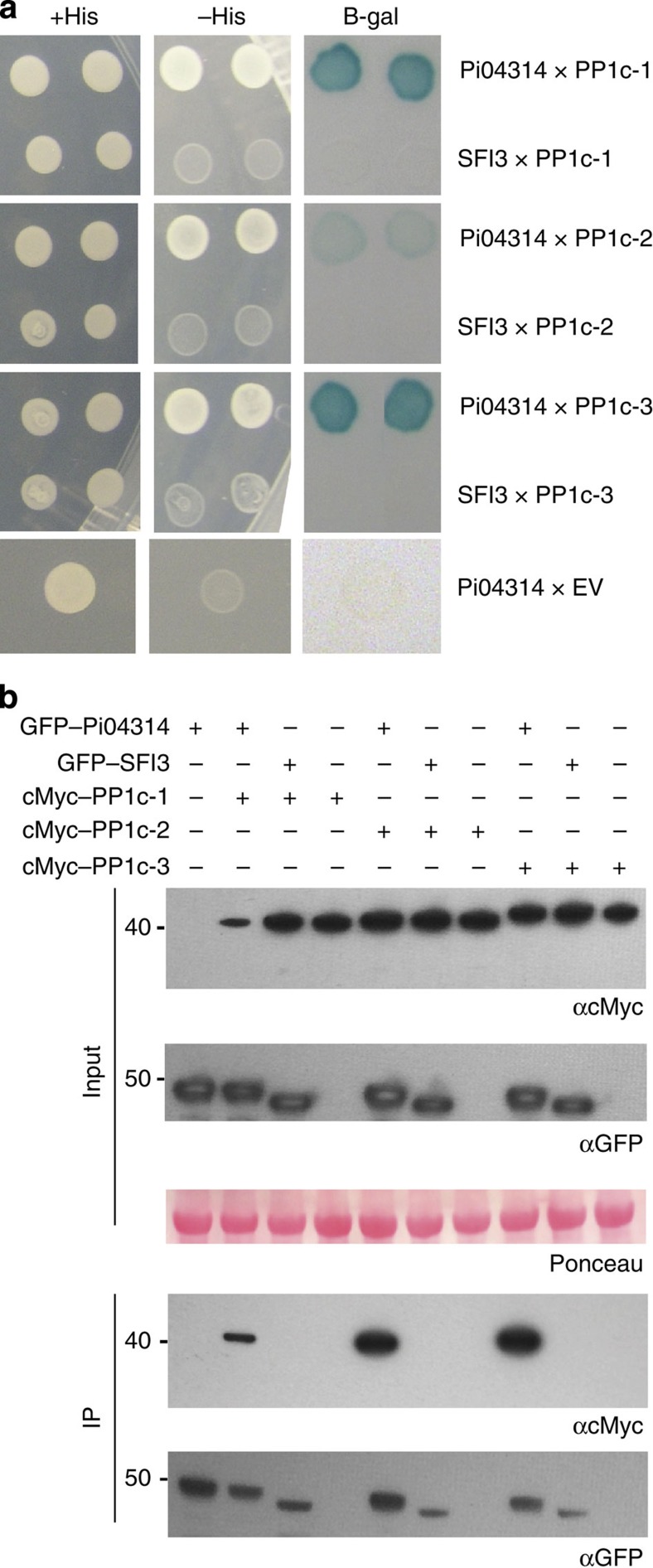
Effector Pi04314 interacts with *Solanum tuberosum* PP1c isoforms in yeast-2-hybrid and immunoprecipitation assays. (**a**) Yeast co-expressing the PP1c isoforms with Pi04314 grew on histidine (HIS) medium and yielded b-galactosidase (B-gal) activity, while those co-expressed with the control SFI3 or empty vector (EV) did not. (**b**) Immunoprecipitation (IP) of protein extracts from agroinfiltrated leaves using GFP–Trap confirmed that cMyc-tagged PP1c isoforms specifically associated with GFP–Pi04314 and not with the GFP–SFI3 control. Expression of constructs in the leaves is indicated by +. Protein size markers are indicated in kDa, and protein loading is indicated by Ponceau stain.

To reconfirm interactions, pairwise Y2H was conducted with the full-length StPP1c prey clones against the Pi04314 bait, using RXLR effector SFI3 as a negative control bait. While SFI3 shows a similar nuclear localization to Pi04314, and also enhances *P. infestans* leaf colonization when transiently expressed *in planta*, unlike Pi04314 it suppresses early transcriptional responses activated by the bacterial PAMP flg22, suggesting that it does not share a similar function^19^. Pi04314 interacts with each PP1c isoform as indicated by induction of B-galactosidase activity and growth on media lacking histidine, whereas the SFI3–PP1c combinations did not activate either reporter (Fig. 2a).

To confirm that these interactions also occur *in planta* co-immunoprecipitation (Co-IP) experiments were conducted using GFP–Pi04314 and N-terminal cMyc-tagged PP1c constructs (cMyc–PP1c-1, cMyc–PP1c-2 and cMyc–PP1c-3), with GFP–SFI3 used as a non-interacting control. The GFP and cMyc fusion constructs were transiently co-expressed in *N. benthamiana* and, following immunoprecipitation using GFP–TRAP_M beads, cMyc–PP1c-1, cMyc–PP1c-2 and cMyc–PP1c-3 constructs were immunoprecipitated in the presence of GFP–Pi04314, but not with the GFP–SFI3 control, whereas all constructs were detected in the relevant input fractions (Fig. 2b).

### Pi04314 re-localizes the StPP1c isoforms from the nucleolus

To investigate the subcellular localization of the StPP1c isoform monomeric red fluorescent protein (mRFP), N-terminal fusion constructs were generated (mRFP–PP1c-1, mRFP–PP1c-2 and mRFP–PP1c-3) and viewed following *Agrobacterium*-mediated expression in *N. benthamiana* using confocal microscopy. Each mRFP–PP1c fusion protein predominantly localized to the nucleoplasm and the nucleolus, with background cytoplasmic fluorescence (Supplementary Fig. 4a).

**Figure 3:**
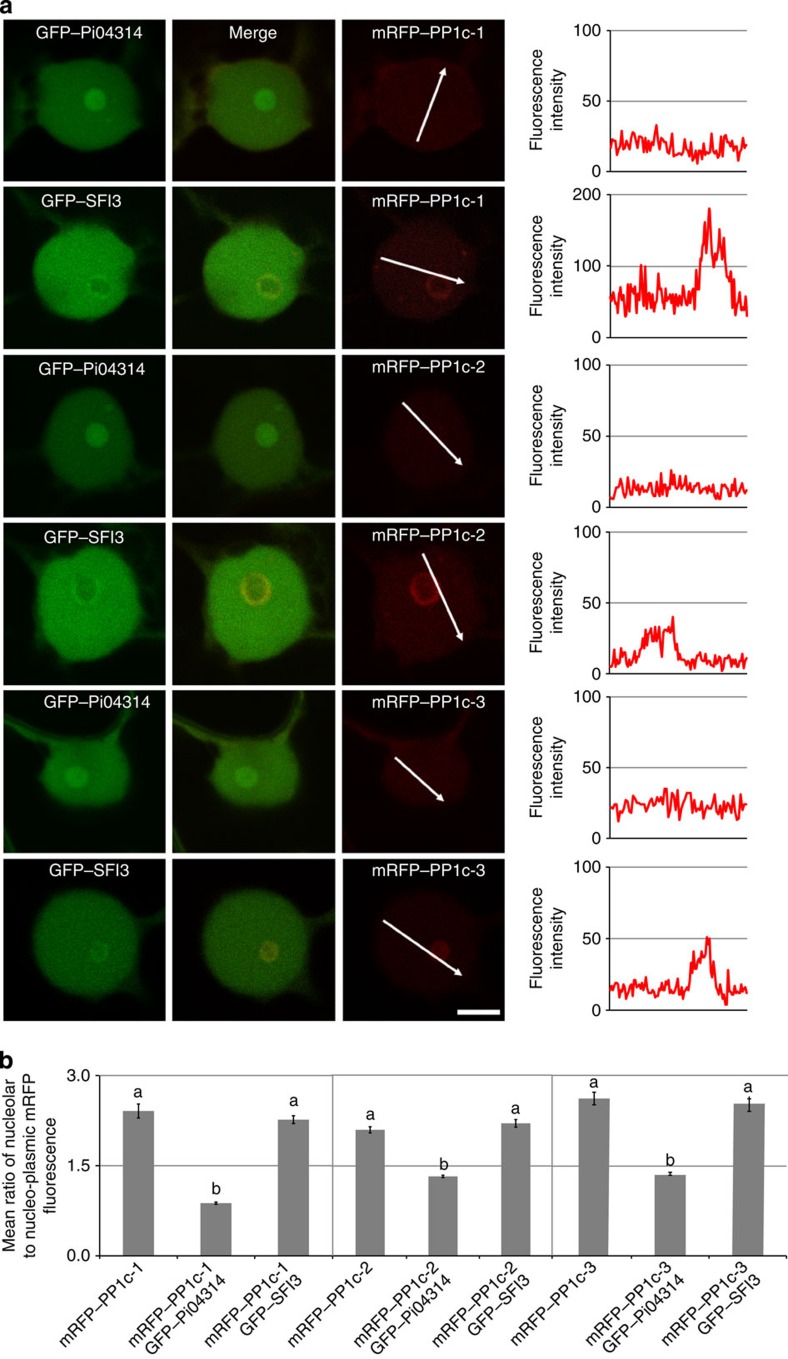
Co-expression of Pi04314 with the PP1c results in reduced nucleolar association of the latter. (**a**) Image sets are single optical sections of typical nuclei co-expressing GFP–Pi04314 or the GFP–SFI3 control with each isoform of PP1c fused to mRFP. Scale bar, 5 μm. White arrows indicate mRFP fluorescence intensity plots shown in graphs to the right of each image. (**b**) Graphs of the average ratios of nucleolar to nucleoplasmic mRFP fluorescence from the PP1c isoforms alone or co-expressed with each effector, demonstrating that significant loss of mRFP–PP1c from the nucleolus occurred only in the presence of GFP–Pi04314. The averages were obtained from images of more than 50 nuclei for each condition. Error bars are s.e. and the graph represents the combined data from six biological replicates. Letters on the graphs denote statistically significant differences (ANOVA, *P*<0.001).

As Pi04314 and StPP1c isoforms displayed similar subcellular localizations when expressed independently, each mRFP–PP1c construct was co-expressed with GFP–Pi04314, or with GFP–SFI3 as a control. Strikingly, each mRFP–PP1c construct displayed reduced nucleolar fluorescence when co-expressed with GFP–Pi04314, whereas there was no change in the nucleolar mRFP–PP1c localization when expressed with GFP–SFI3 (Fig. 3a).

The ratio of mRFP–PP1c nucleolar to nucleoplasmic fluorescence was measured in more than 50 nuclei for each construct and (co-)expression event, and indeed revealed a statistically significant decrease (ANOVA, *P*<0.001) when co-expressed with GFP–Pi04314, compared with co-expression with GFP–SFI3, or when each mRFP–PP1c construct was expressed alone (Fig. 3b). The reduced nucleolar mRFP–PP1c accumulation in the presence of GFP–Pi04314 may be explained by (1) Pi04314 interaction causes re-localization of PP1c out of the nucleolus; or (2) Pi04314 provokes the degradation of PP1c specifically in the nucleolus. However, there was no evidence of differential mRFP–PP1c accumulation when expressed alone or co-expressed with GFP–Pi04314 or GFP–SFI3 (Supplementary Fig. 4b). In addition, a decrease in nucleoplasmic GFP fluorescence was also specifically observed when GFP–Pi04314 was co-expressed with each mRFP–PP1c isoform, compared with GFP–Pi04314 alone (Supplementary Fig. 5). In the absence of detectable protein degradation, the observed decrease in both interacting partners in the nucleolus is consistent with GFP–Pi04314/mRFP–PP1c complexes being re-localized from the nucleolus, potentially to the nucleoplasm. Consistent with this, when co-expressed with the mis-localized _myr_GFP–Pi04314, mRFP–PP1c nucleolar fluorescence was unperturbed (Supplementary Fig. 6). In contrast, as anticipated, when mRFP–PP1c-1 was co-expressed with the nuclear-focused _NLS_GFP–Pi04314, reduced nucleolar fluorescence relative to nucleoplasmic fluorescence was observed for both effector and putative target (Supplementary Fig. 7).

**Figure 4:**
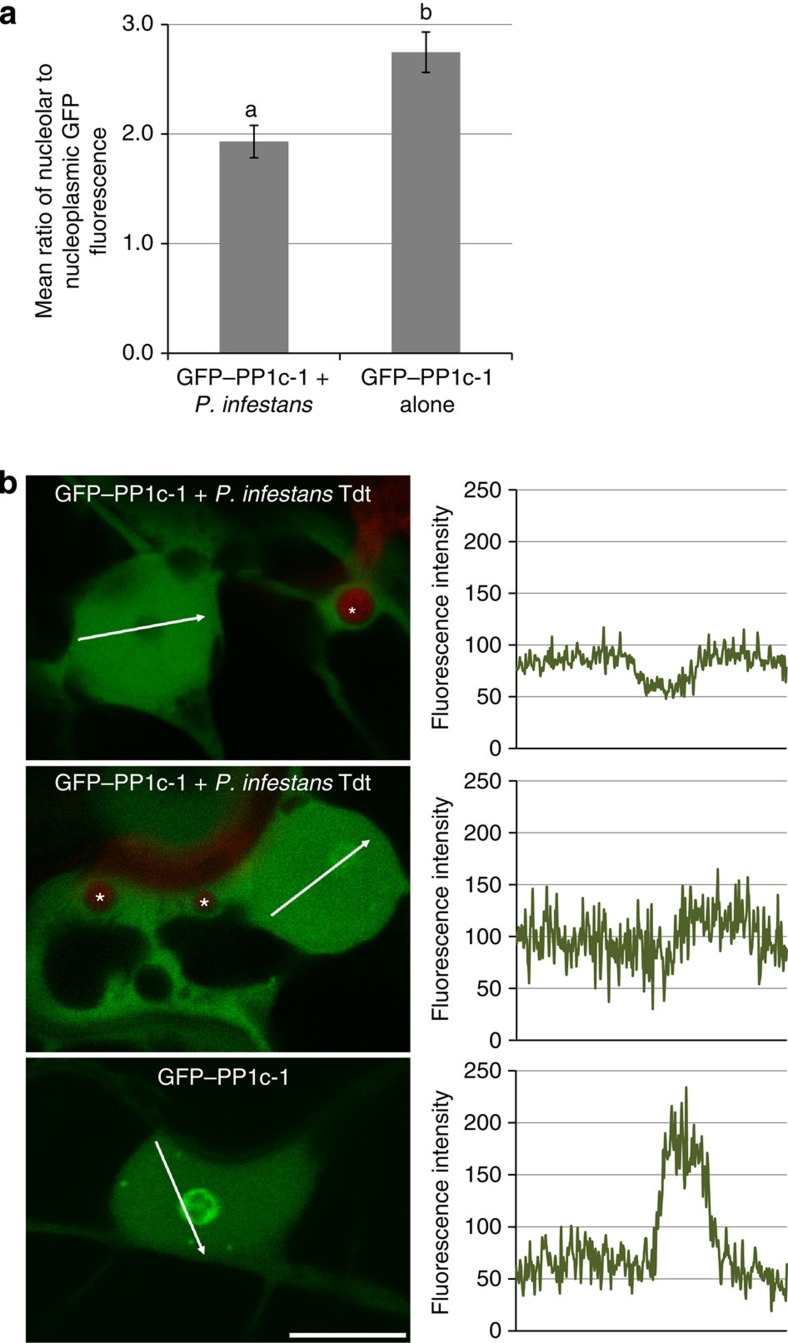
Nucleolar accumulation of PP1c-1 is reduced in nucleoli of the cell interacting with *P. infestans* haustoria. (**a**) Graph showing that mean ratio of nucleolar to nucleoplasmic fluorescence of GFP–PP1c-1 is reduced in cells containing *P. infestans* haustoria (combined from three biological replicates with a total of 35 nuclei measured from cells expressing GFP–PP1c-1 fluorescence and showing red fluorescent haustoria from *P. infestans* strain 88069 tdt), compared with 22 GFP–PP1c-1-expressing nuclei in uninfected cells. Letters denote statistical significance (ANOVA, *P*=0.001). (**b**) Images are representative of the different patterns observed in GFP–PP1c-1 re-localization from the nucleolus. Nucleolar GFP–PP1c-1 fluorescence in haustoriated cells was either considerably attenuated (upper panel) or reduced (middle panel), compared with uninfected cells (lower panel). Corresponding fluorescence intensity plots (from arrowed lines indicated in each image) are shown in graphs to the right of the image. Red fluorescent haustoria are indicated with *; scale bar, 10 μm.

To investigate whether re-localization of PP1c from the nucleolus occurs also during infection, *N. benthamiana* expressing GFP–PP1c-1 was inoculated with zoospores of transgenic *P. infestans* 88069 expressing the fluorescent protein Td-tomato. The ratio of nucleolar to nucleoplasmic GFP fluorescence was significantly reduced in host cells interacting with haustoria, compared with uninfected *N. benthamiana* leaf cells (Fig. 4). This indicates that re-localization of GFP–PP1c-1 also occurs during early stages of infection.

### Pi04314 interacts with PP1c via a conserved R/KVxF motif

Protein phosphatase activity is often regulated through interactions with protein-binding partners. PP1c phosphatases in mammals associate with >200 regulatory subunits, which target PP1c to different sites within the cell and provide substrate specificity. Most regulatory subunits interact with PP1c through a conserved PP1c-binding motif known as the R/KVxF, which conforms to the consensus [K/R][K/R][V/I]X[F/W]. The R/KVxF-binding groove of PP1c is 20 Å away from the active site, and thus binding does not inhibit phosphatase activity^29^. Nevertheless, many inhibitory subunits of PP1c also interact via the R/KVxF motif, as exemplified by the widely conserved inhibitor-2 subunit in *Arabidopsis*^30^. We observed a candidate motif, KVTF, from residues 117 to 120 in the C-terminal region of Pi04314, raising the possibility that this mediates interaction between the effector and PP1c isoforms. We thus mutated these residues to alanines (Supplementary Fig. 8) to create Pi04314mut.

**Figure 5:**
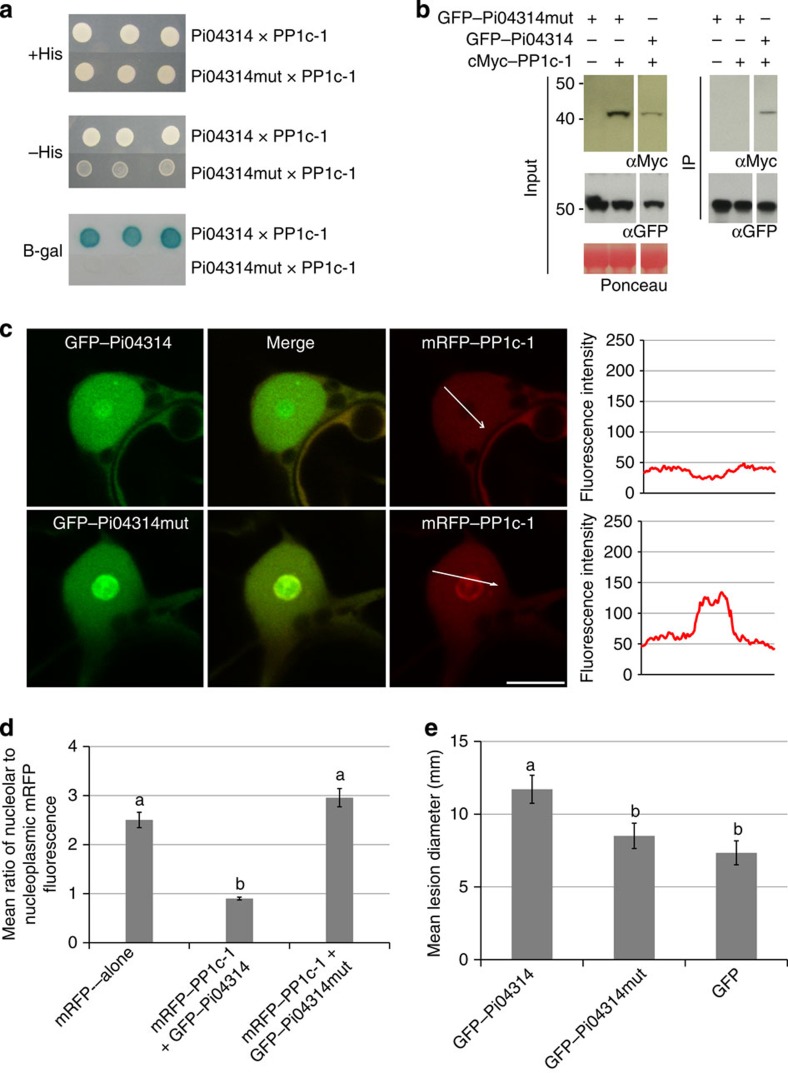
Mutation of the PP1c-binding motif in Pi04314 results in a loss of PP1c association. (**a**) Yeast-2-hybrid assay following co-expression of PP1c-1 and wild-type (WT) Pi04314, which grew on −histidine (−HIS) medium and had β-galactosidase (B-gal) activity, while co-expression of PP1c-1 with Pi04314mut did not. (**b**) Immunoprecipitation of WT GFP–Pi04314 and GFP–Pi04314mut protein extracts from agroinfiltrated leaves using GFP–Trap confirmed that cMyc–PP1c-1 co-immunoprecipitated only with WT GFP–Pi04314. Expression of constructs in the leaves is indicated by +. Protein size markers are indicated in kDa, protein loading is indicated by Ponceau stain, and antibodies used are as indicated (αcMyc and αGFP). (**c**) Single optical section through co-expressing nuclei show that the mutated effector fusion GFP–Pi04314mut co-expressed with mRFP–PP1c-1 did not cause reduction of mRFP fluorescence in the nucleolus, whereas the WT GFP–Pi04314 did. Scale bar, 10 μm. White arrows indicate mRFP fluorescence intensity plots shown in graphs to the right of each image. (**d**) Graph shows the average ratio of nucleolar to nucleoplasmic mRFP fluorescence from the mRFP–PP1c-1 expressed alone, with the WT effector fusion GFP–Pi04314 and with the mutated effector GFP–Pi04314mut. The averages were obtained from a minimum of 30 nuclei for each sample. Error bars are s.e. and the graph represents the combined data from three biological replicates. Letters on the graph denote statistically significant differences (ANOVA, *P*<0.001). (**e**) The GFP fusion to the mutated Pi04314 is no longer able to significantly promote *P. infestans* infection as measured by lesion diameter. Error bars are s.e. and the graph represents the combined data from three biological replicates (*n*=108 per construct). Letters on the graph denote statistically significant differences (ANOVA, *P*≤0.022).

Pi04314mut was unable to interact with PP1c-1, PP1c-2 or PP1c-3 in yeast, using the Y2H assay (shown for PP1c-1; Fig. 5), or *in planta* using Co-IP (Fig. 5; Supplementary Fig. 9a). Moreover, it failed to re-localize the PP1c isoforms from the nucleolus (Fig. 5c,d; Supplementary Fig. 9b, c). Critically, unlike the wild-type (WT) Pi04314, it failed to enhance *P. infestans* colonization of *N. benthamiana* leaves (Fig. 5e). Pi04314 interaction with PP1c isoforms thus mimics regulatory subunits, in that it binds via an R/KVxF motif. Three possibilities present themselves with respect to the potential mode of action of this effector: (1) it acts as an inhibitory subunit to prevent PP1c activity; (2) it competes for the binding of endogenous PP1c regulatory subunits, thus indirectly acting as an inhibitor of normal PP1c activity; or (3) it acts as a regulatory subunit, targeting PP1c activity to defined substrates for dephosphorylation.

### Silencing of *NbPP1c* attenuates *P. infestans* infection

To test the potential role of PP1c activity in preventing or promoting disease, we identified the equivalent *PP1c* family members in *N. benthamiana* (Supplementary Fig. 2) and silenced their expression using VIGS. Because phosphatase-encoding portions of PP1c family members are highly conserved, we selected the divergent portions at the terminal 5′ and 3′ regions from each gene, *NbPP1c-1*, *NbPP1c-2* and *NbPP1c-3*, and synthesized two DNA strands for cloning into the tobacco rattle virus (TRV) VIGS vector: one combining the three 5′ ends (TRV–PP1c5′), and the other combining the three 3′ ends (TRV–PP1c3′; Supplementary Fig. 10a). *Agrobacterium* strains containing these constructs were infiltrated into 2-week-old *N. benthamiana* seedlings. Gene silencing levels were checked after 2–3 weeks in plants in each biological replicate using qRT–PCR. Both TRV–PP1c5′ and TRV–PP1c3′ VIGS constructs consistently knocked down *NbPP1c-1*, *NbPP1c-2* and *NbPP1c-3* transcript levels by 80–90% compared with transcript accumulation in TRV–GFP control plants (Supplementary Fig. 10b). Although slightly smaller than plants expressing TRV–GFP, the growth and development of plants expressing TRV–PP1c5′ and TRV–PP1c3′ was not otherwise perturbed (Supplementary Fig. 10c). To investigate potential off-target silencing, transcript levels were determined for an additional *PP1c* gene with reciprocal best BLAST hits in potato and *N. benthamiana*, termed *StPP1c-4* and *NbPP1c-4*, respectively, which are similar to both AtTOPP8 and AtTOPP9 from *Arabidopsis* (Supplementary Fig. 2). StPP1c-4 does not interact with Pi04314 in yeast, using Y2H, or *in planta* using Co-IP (Supplementary Fig. 11a, b), suggesting that it is not a target of this effector. We found that *NbPP1c-4* transcript levels were not reduced by TRV–PP1c5′ or TRV–PP1c3′ expression in *N. benthamiana* (Supplementary Fig. 10b), indicating that our silencing is specific to *NbPP1c-1*, *NbPP1c-2* and *NbPP1c-3*.

**Figure 6:**
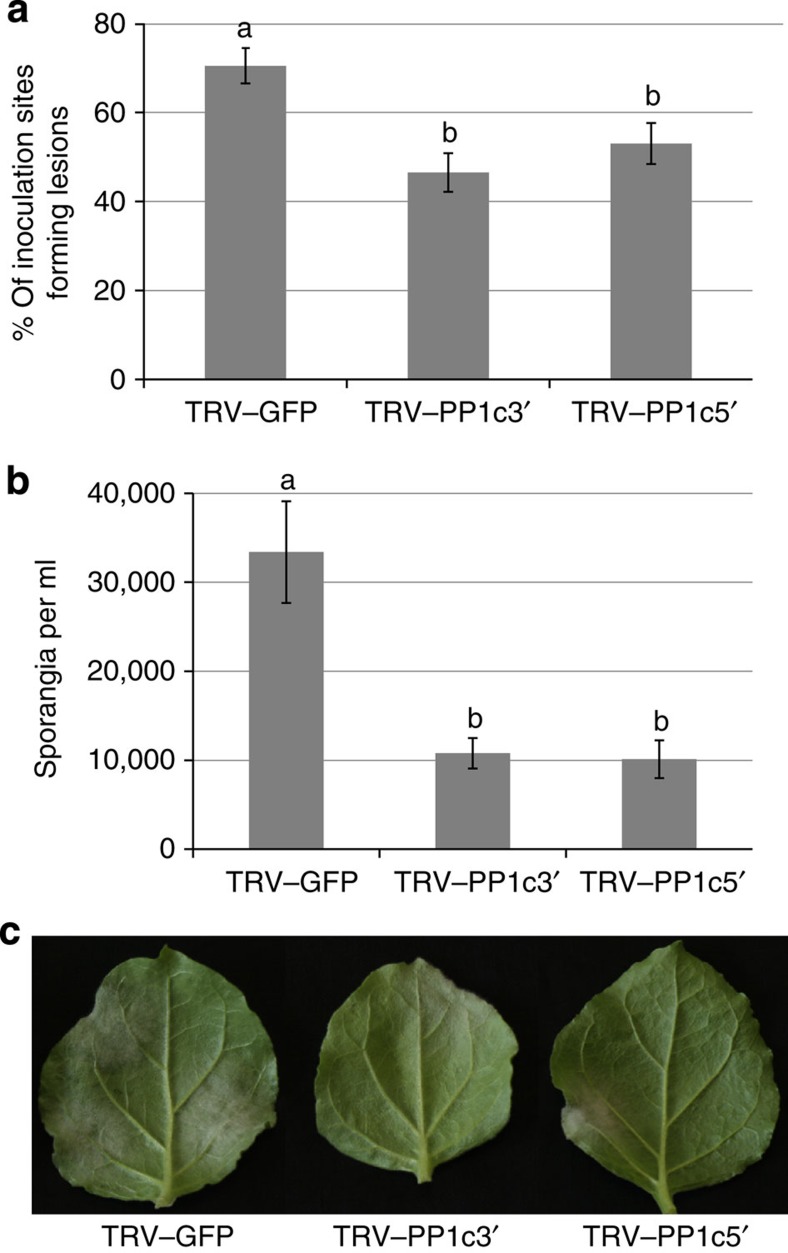
Virus-induced gene silencing of the *NbPP1c* isoforms reduces *P. infestans* leaf colonization. (**a**) Graph showing the reduction in percentages of inoculations resulting in *P. infestans* lesions in plants expressing TRV–PP1c3′ or TRV–PP1c5′, compared with a TRV–GFP control. Error bars are s.e. and the graph represents the combined data from six biological replicates (*n*=50 per construct). Letters on the graph denote statistically significant differences (ANOVA, *P*≤0.009). (**b**) Graph showing the reduction in the average numbers of sporangia per ml recovered from infected leaves of plants expressing TRV–PP1c3′ or TRV–PP1c5′, compared with the TRV–GFP control plants. Error bars are s.e. and the graph represents the combined data from four biological replicates (*n*=32 per construct). Letters on the graph denote statistically significant differences (ANOVA, *P*<0.001). (**c**) Example leaves showing *P. infestans* lesion development on control and TRV–PP1c3′ or TRV–PP1c5′ plants.

We investigated whether *P. infestans* colonization was altered by silencing *NbPP1c-1*, *NbPP1c-2* and *NbPP1c-3*. Remarkably, in *N. benthamiana* plants expressing either TRV–PP1c5′ or TRV–PP1c3′, significantly fewer *P. infestans* infection lesions developed compared with TRV–GFP control plants. Moreover, on leaves where lesions formed, significantly fewer *P. infestans* sporangia were produced (Fig. 6). This indicates that *P. infestans* colonization of the silenced plants was attenuated, suggesting that the pathogen requires PP1c for disease development. This prompted us to investigate whether PP1c activity is beneficial to *P. infestans* infection.

### Phosphatase-dead PP1c–1mut attenuates infection

As the silencing experiments suggested that PP1c isoforms may be required for full virulence of *P. infestans*, we deduced that the KVTF-mediated interaction with Pi04314 does not conform with the effector acting as (1) an inhibitor of PP1c activity, or (2) indirectly inhibiting PP1c functions by competing for binding with endogenous regulatory subunits, although this indeed may be a partial consequence of interaction. Instead, we propose that Pi04314 interacts with PP1c isoforms to use their activity.

**Figure 7:**
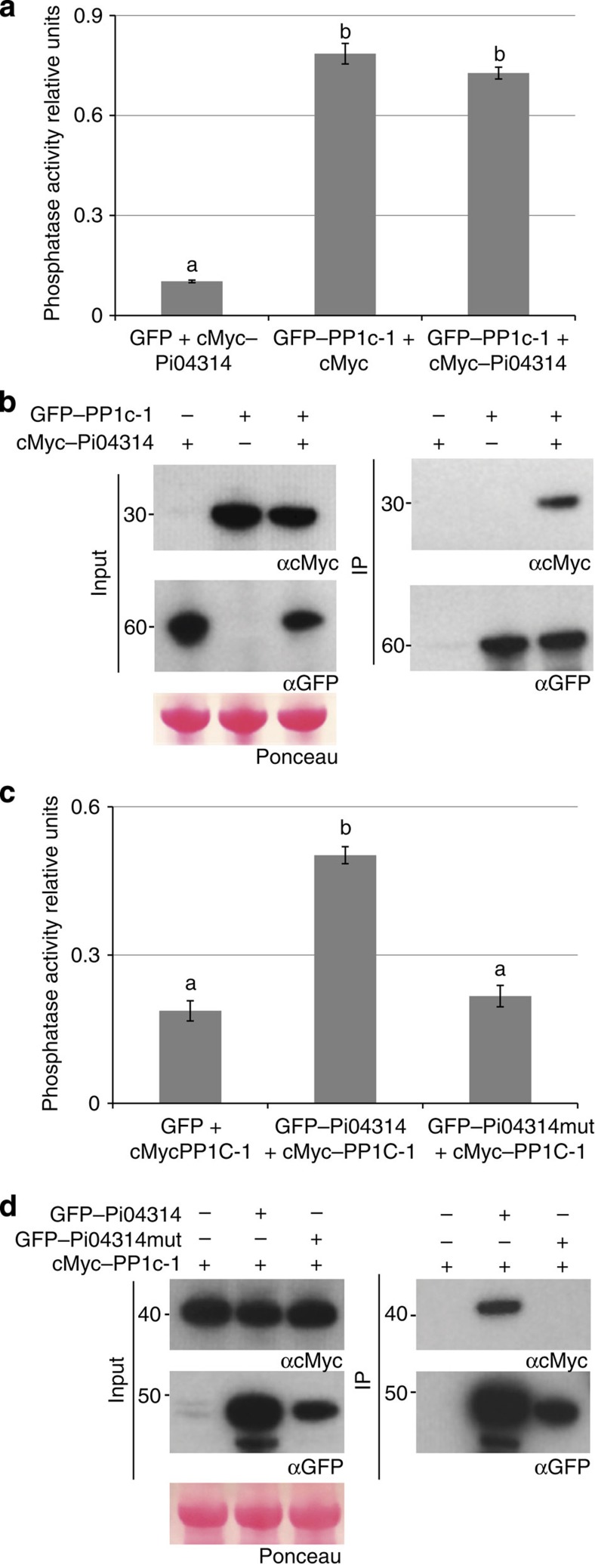
Pi04314 does not inhibit PP1c-1 phosphatase activity. (**a**) Graph of phosphatase activity measured directly on GFP–Trap_M beads following immunoprecipitation of GFP co-expressed with cMyc–Pi04314; or GFP–PP1c-1 co-expressed with either cMyc vector or cMyc–Pi04314. Error bars are s.e. and the graph represents combined data from three biological replicates. Letters on the graph denote statistically significant differences (ANOVA, *P*<0.001). (**b**) Immunoprecipitation of protein extracts from agroinfiltrated leaves using GFP–Trap confirmed that cMyc–Pi04314 is co-immunoprecipitated with GFP–PP1c-1. Expression of constructs in the leaves is indicated by +. Protein size markers are indicated in kDa, and protein loading is indicated by Ponceau stain. Antibodies used are indicated (αcMyc and αGFP). (**c**) Graph of phosphatase activity measured directly on GFP–Trap_M beads following immunoprecipitation of GFP, GFP–Pi04314 or GFP–Pi04314mut co-expressed with cMyc–PP1c-1. Error bars are s.e. and the graph represents the combined data from three biological replicates. Letters on the graph denote statistically significant differences (ANOVA, *P*<0.001). (**d**) Immunoprecipitation of protein extracts from agroinfiltrated leaves using GFP–Trap confirmed the cMyc–PP1c-1 co-immunoprecipitated with the GFP–Pi04314 effector, but not with GFP–Pi04314mut. Expression of constructs in the leaves is indicated by +. Protein size markers are indicated in kDa, and protein loading is indicated by Ponceau stain. Antibodies used are as indicated (αcMyc and αGFP).

Before testing this, we investigated whether Pi04314 could act as an inhibitor of PP1c activity. Initially, we co-expressed GFP–PP1c-1 with the cMyc empty vector or with cMyc–Pi04314 and, as a control, co-expressed GFP with cMyc–Pi04314. Following immunoprecipitation with GFP–Trap_M beads, phosphatase activity was directly assayed on the beads before elution of proteins for western analyses. Whereas GFP expressed with cMyc–Pi04314 provided no phosphatase activity, similar high levels (ANOVA, *P*=0.001) of activity were detected with GFP–PP1c-1 expressed with either cMyc empty vector or with cMyc–Pi04314 (Fig. 7a). Immunoblots indicated that cMyc–Pi04314 was co-immunoprecipitated, as anticipated, only with GFP–PP1c-1 (Fig. 7b). This indicates that cMyc–Pi04314 in complex with GFP–PP1c-1 does not inhibit phosphatase activity. Reciprocally, cMyc–PP1c-1 was co-expressed with free GFP, with GFP–Pi04314 or with non-interacting GFP–Pi04314mut. Each GFP fusion protein was immunoprecipitated with GFP–Trap_M beads and phosphatase activity again directly assayed on the beads. Whereas phosphatase activity was detected in the case of GFP–Pi04314, no such activity was observed with free GFP or GFP–Pi04314mut (Fig. 7c). Following elution of proteins from the beads, we confirmed that cMyc–PP1c-1 was co-immunoprecipitated only in the presence of GFP–Pi04314 (Fig. 7d). Thus, Pi04314 associated with PP1c-1 does not inhibit phosphatase activity.

**Figure 8:**
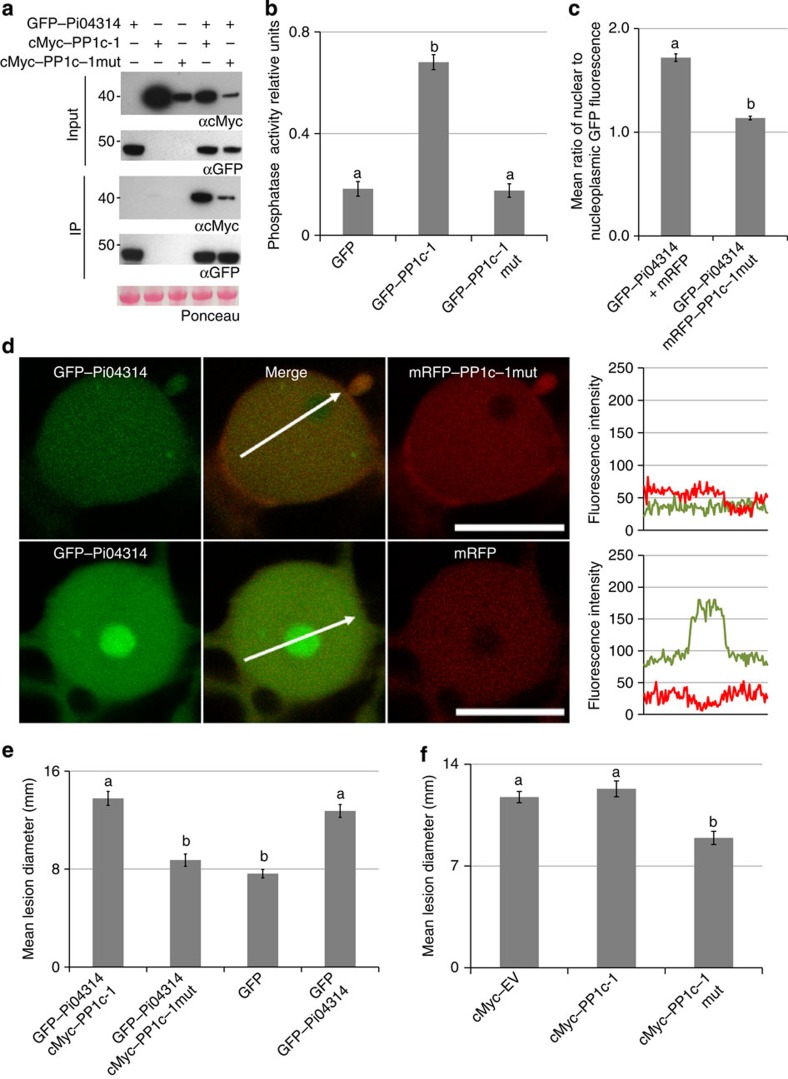
A phosphatase-dead mutant of PP1c-1 reduced *P. infestans* leaf colonization, and the enhanced colonization promoted by expression of the effector. (**a**) Immunoprecipitation of protein extracts from agroinfiltrated leaves using GFP–Trap. Expression of constructs in the leaves is indicated by +. Protein size markers are indicated in kDa, and protein loading is indicated by Ponceau stain. Antibodies used are as indicated (αcMyc and αGFP). (**b**) Graph of the phosphatase activity of the wild-type GFP–PP1c-1 and mutated GFP–PP1c–1mut showing that the mutant showed similar background activity to the GFP empty vector control. Error bars are s.e. and the graph represents the combined data from three biological replicates. Letters on the graph denote statistically significant differences (ANOVA, *P*<0.001). (**c**) Graph of the average ratio of nucleolar to nucleoplasmic GFP fluorescence from the GFP–Pi04314 fusion protein expressed with mRFP–PP1c–1mut or, as a control, free mRFP. Error bars are s.e. and the graph represents the combined data from three biological replicates (*n*=40 per construct). Letters on the graph denote statistically significant differences (ANOVA, *P*<0.001). (**d**) Single optical section through co-expressing nuclei show that the effector fusion GFP–Pi04314 was reduced in the nucleolus when co-expressed with mRFP–PP1c–1mut, but not with free mRFP. Scale bar, 10 μm. White arrows indicate mRFP fluorescence intensity plots shown in graphs to the right of each image. (**e**) Co-expression of the cMyc–PP1c-1 wild-type did not significantly change the enhancement of *P. infestans* growth caused by expression of the GFP–Pi04314 effector compared with the control. However, co-expression of the mutant cMyc–PP1c–1mut with GFP–Pi04314 caused a significant reduction in effector-induced enhancement of colonization. Error bars are s.e. and the graph represents the combined data from three biological replicates (*n*=102 per construct). Letters on the graph denote statistically significant differences (ANOVA, *P*<0.001). (**f**) Expression of wild-type cMyc–PP1c-1 did not significantly alter *P. infestans* infection compared with the control empty (cMyc) vector (EV), but expression of the phosphatase-dead mutant cMyc–PP1c–1mut significantly reduced lesion growth. Error bars are s.e. and the graph represents the combined data from three biological replicates (*n*=73 per construct). Letters on the graph denote statistically significant differences (ANOVA, *P*<0.001).

We hypothesized that overexpression of a phosphatase-dead mutant of any one of the PP1c isoforms could act as ‘dominant negative', preventing the enhanced *P. infestans* leaf colonization by binding to the effector but no longer providing phosphatase activity. On the basis of previous characterization of the active-site residue in PP1c isoforms^31^, we identified the equivalent residue in PP1c-1 (H129; Supplementary Fig. 3b) and mutated it to an alanine, generating PP1c–1mut. Following expression in *N. benthamiana*, free GFP, GFP–PP1c-1 and GFP–PP1c–1mut were immunoprecipitated using GFP–TRAP_M beads, demonstrating each fusion protein was stable and intact (Supplementary Fig. 12a), and phosphatase activity was directly assayed on the beads. Whereas GFP–PP1c-1 phosphatase activity was statistically significantly (ANOVA, *P*<0.001) higher than the GFP control, no such activity was detected with the GFP–PP1c–1mut, confirming its inactivity (Fig. 8a; Supplementary Fig. 12b).

For PP1c–1mut to act as dominant negative, its interaction with Pi04314 must be maintained. We co-expressed cMyc–PP1–1cmut or cMyc–PP1c-1 with GFP–Pi04314, immunoprecipitating the latter with GFP–TRAP_M beads, and observed that cMyc–PP1–1cmut, like cMyc–PP1c-1, was co-immunoprecipitated with the effector (Fig. 8b). This confirmed that the H129A mutation did not perturb interaction with Pi04314. When we investigated the localization of mRFP–PP1c–1mut using confocal microscopy, we observed that, like the WT protein, it accumulated in the nucleoplasm with cytoplasmic background. However, unlike the WT protein, there was no detectable accumulation of mRFP–PP1c–1mut in the nucleolus (Supplementary Fig. 12). Nevertheless, consistent with the interaction of Pi04314 with PP1c–1mut being maintained *in planta*, co-expression of mRFP–PP1c–1mut with GFP–Pi04314 still significantly reduced accumulation of the effector in the nucleolus, compared with a free mRFP control, which also does not localize in the nucleolus (Fig. 8c,d).

To investigate whether the PP1c–1mut had an effect on the enhanced *P. infestans* leaf colonization promoted by transient expression of Pi04314, free GFP was expressed on one-half of *N. benthamiana* leaves and either GFP–Pi04314 alone, GFP–Pi04314 co-expressed with WT cMyc–PP1c-1, or GFP–Pi04314 co-expressed with cMyc–PP1c–1mut on the other half. *P. infestans* zoospores were drop-inoculated onto each half of the leaves, and infection allowed to progress for 6 days. Whereas GFP–04314 alone, as anticipated, or GFP–Pi04314 co-expressed with cMyc–PP1c-1 WT, promoted similar levels of significantly enhanced (ANOVA, *P*<0.001) *P. infestans* lesion development compared with the GFP control, lesion sizes on leaves where GFP–04314 was co-expressed with cMyc–PP1c–1mut were no different to the GFP control (Fig. 8e). This indicates that the expression of the phosphatase-dead PP1c–1mut form prevents Pi04314 effector activity.

To further investigate the potential for the phosphatase-dead PP1c–1mut form to be detrimental to *P. infestans* infection, an empty cMyc vector (EV) was expressed on one-half of *N. benthamiana* leaves and either cMyc–PP1c-1 WT or cMyc–PP1c–1mut expressed on the other. A higher inoculum of *P. infestans* zoospores was drop-inoculated onto each side of the *N. benthamiana* leaves to promote enhanced colonization, and infections were allowed to progress for 7–8 days. Lesion sizes on leaf halves where cMyc–PP1c WT was expressed were no different to those where EV control was expressed. In contrast, expression of cMyc–PP1c–1mut resulted in significantly (ANOVA, *P*<0,001) reduced lesion sizes compared with the EV control (Fig. 8f). Thus, overexpression of the phosphatase-dead PP1c–1mut reduces levels of *P. infestans* infection.

### Pi04314 suppresses JA- and SA-responsive genes

We have shown that when expressed *in planta*, Pi04314 interacts with three isoforms of PP1c to enhance *P. infestans* leaf colonization in a phosphatase activity-dependent manner. However, it remains unclear that how the effector could be of benefit to *P. infestans*. We investigated whether Pi04314 could suppress cell death triggered by the *P. infestans* PAMP INF1, or by co-expression of the tomato cell-surface receptor Cf4 and *Cladosporium fulvum* effector AVR4. We have shown previously that Cf4-mediated cell death is suppressed by the RXLR effector PexRD2 (ref. 20) and that cell death mediated by both INF1 and Cf4 is suppressed by AVR3a^16^. In contrast to GFP–AVR3a, GFP–Pi04314 failed to suppress either INF1- or Cf4-mediated cell death (Supplementary Fig. 13).

**Figure 9:**
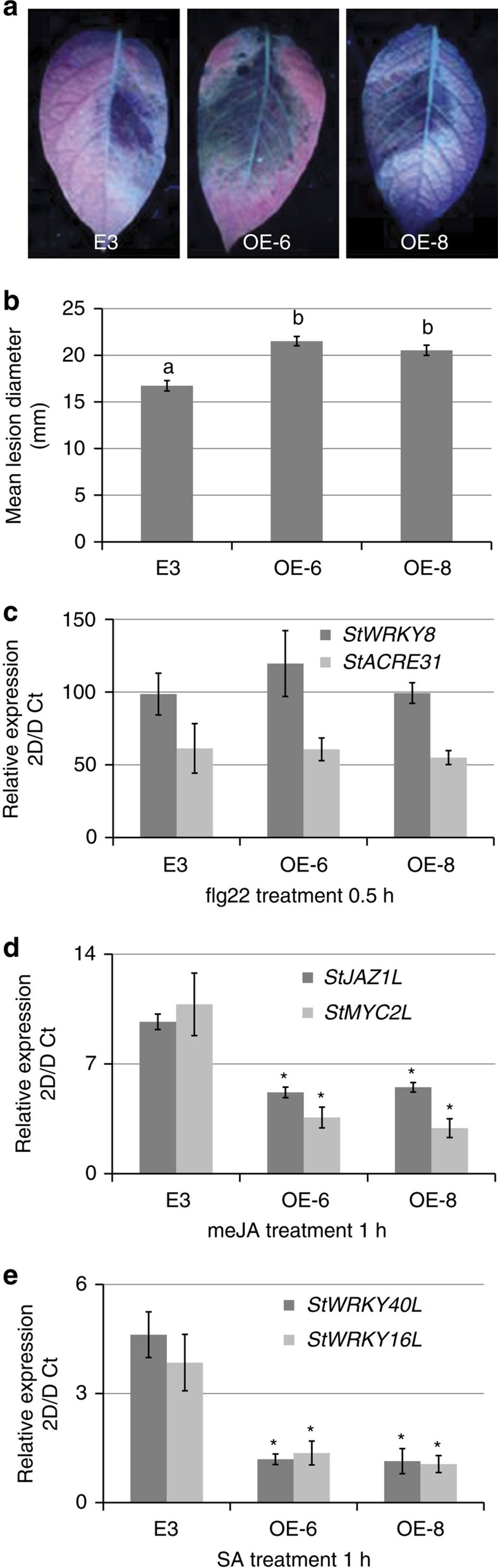
Transgenic potato expressing Pi04314 enhances *P. infestans* leaf colonization and attenuates induction of JA- and SA-responsive genes. (**a**) Representative leaf images, exposed to UV, showing increased lesion sizes on Pi04314-expressing transgenic lines E-6 and OE-8, compared with the untransformed control, cv E3. (**b**) Mean lesion diameter is significantly increased in OE-6 and OE-8 lines compared with the control, cv E3. Letters denote statistical significance (ANOVA, *P*<0.001) from three biological replicates, each containing inoculation of three leaves from each of the six plants. (**c**) Relative expression of flg22 marker genes *StWRKY8* and *StACRE31* 30 min after treatment with flg22 in E3, OE-6 and OE-8 lines, compared with untreated lines (which was given a value of 1). (**d**) Relative expression of JA-responsive genes *StJAZ1-like* (*StJAZ1L*) and *StMYC2L* 1 h after treatment with meJA in E3, OE-6 and OE-8 lines, compared with untreated lines (which was given a value of 1). (**e**) Relative expression of SA-responsive genes *StWRKY40-like* (*StWRKY40L*) and *StWRKY16L* 1 h after treatment with SA in E3, OE-6 and OE-8 lines, compared with untreated lines (which was given a value of 1). Results in B-E are the mean of three independent biological replicates. Error bars show s.e. and * denotes significantly reduced induction (ANOVA, *P*<0.001) of responsive genes in transgenic lines compared with the E3 control.

We next generated transgenic susceptible potato cultivar E3 lines expressing Pi04314 and selected two lines (OE-6 and OE-8) for study (Supplementary Fig. 14). Both lines showed enhanced *P. infestans* colonization (Fig. 9a,b) relative to untransformed cv E3, consistent with the observation (Fig. 1) that transient expression of Pi04314 enhanced colonization in *N. benthamiana*. We treated leaves of the control cv E3, OE-6 and OE-8 with flg22 and investigated early-responsive gene *WRKY8*, encoding a transcription factor that is directly phosphorylated by the MAPK salicylic acid-induced protein kinase (SIPK)^32^ and *ACRE31*, a generally used early flg22 marker^26^. Both genes were strongly upregulated in cv E3, OE-6 and OE-8 only 30 min after flg22 treatment (Fig. 9; Supplementary Fig. 14). This agrees with the previous observation that Pi04314 does not suppress early flg22-responsive gene induction^19^. We next selected genes that rapidly respond to exogenous application of SA and methyl jasmonate (meJA), based on recent potato microarray studies^33^, which are available on a searchable database (https://ics.hutton.ac.uk/solarray/). We selected two genes upregulated at 1 h after treatment of meJA (*StMYC2-like* and *StJAZ1-like*), and two genes upregulated at 1 h by SA (designated *StWRKY40-like* and *StWRKY16-like*) and confirmed their upregulation at 1 h in potato cv E3 following appropriate treatments (Fig. 9; Supplementary Fig. 14). In contrast, following treatments with SA or meJA, induction of all four genes was attenuated in lines OE-6 and OE-8 (Fig. 9), suggesting that Pi04314 may attenuate SA and JA defence pathways.

## Discussion

*P. infestans* effector PITG_04314/PexRD24 (Pi04314) is a core RXLR effector that is expressed specifically in the biotrophic phase of infection in all pathogen isolates tested^22^,^23^,^24^. Although future work to silence expression of *Pi04314* in *P. infestans* may reveal whether this effector is essential for infection, we show that its transient expression in leaves of the model late blight host plant *N. benthamiana*, and stable transgenic expression in potato, both result in enhanced *P. infestans* leaf colonization, supporting its role as an effector. Moreover, Pi04314 attenuates upregulation of genes responding early to SA and meJA, suggesting that it may suppress host defence pathways. Pi04314 localizes to the host nucleus and nucleolus, and this localization is required and sufficient for enhanced pathogen colonization. It interacts in yeast and *in planta* with three PP1c isoforms (termed PP1c-1, PP1c-2 and PP1c-3) and both they and the effector are apparently re-localized from the nucleolus, presumably to the nucleoplasm. Critically, GFP–PP1c-1 nucleolar fluoresence is also reduced during the biotrophic stage of infection. Pi04314 contains an R/KVxF motif (KVTF), and this is required for PP1c interaction. Mutation of the KVTF sequence abolishes interaction with, and re-localization of, PP1c isoforms and prevents enhancement of *P. infestans* leaf colonization, indicating that Pi04314 mimics regulatory subunits in its interaction with PP1c. Silencing of all three *PP1c* isoforms or overexpression of a phosphatase-dead PP1c-1 (PP1c–1mut) attenuates infection, demonstrating that host PP1c activity is required for full disease development. Moreover, co-expression of PP1c–1mut with Pi04314 abolishes the enhancement of *P. infestans* leaf colonization provided by the effector. We discuss these points below, and argue that PP1c isoforms are susceptibility factors that are utilized by Pi04314 to promote late blight disease.

GFP–Pi04314 transient expression inside plant cells promoted a significant enhancement to *P. infestans* leaf colonization, indicating that the activity of the effector benefits infection. GFP–Pi04314 was localized primarily in the nucleolus and nucleoplasm, with faint cytoplasmic fluorescence. The beneficial activity of the effector was considerably reduced when it was directed away from the nucleus with a myristoylation signal, suggesting that the nucleus is an important site of Pi04314 action. Taken alone, this observation would not rule out a functional role for Pi04314 that involved shuttling between the cytoplasm and the nucleus. However, when Pi04314 was focused in the nucleus with an NLS, the beneficial activity of the effector was not diminished. While background levels of the _NLS_GFP–Pi04314 fusion may yet remain in the cytoplasm, we argue that the lack of a statistically significant alteration in the contribution of the effector to enhanced *P. infestans* colonization suggests that a cytoplasmic phase is not critical for Pi04314 function (Fig. 1). We therefore conclude that Pi04314 activity resides primarily in the nucleus. This is supported by the identification of three host PP1c isoforms as interactors of Pi04314 (Fig. 2), all of which also localize predominantly in the nucleolus and nucleus when expressed as mRFP fusions. However, when GFP–Pi04314 and mRFP–PP1c isoforms are co-expressed, fluorescence from both fusion proteins is reduced in the nucleolus (Fig. 3; Supplementary Fig. 5). Given that there is no apparent reduction in the protein levels of mRFP–PP1c (Supplementary Fig. 4b), we propose that the Pi04314/PP1c complex is re-localized from the nucleolus, rather than degraded. Furthermore, as the activity of the effector, in terms of enhancing *P. infestans* leaf colonization when transiently expressed in *N. benthamiana*, is not diminished when Pi04314 is focused in the nucleus with an NLS, we propose that the GFP–Pi04314/mRFP–PP1c complex is re-localized from the nucleolus to the nucleoplasm, rather than outside of the nucleus.

Ser/thr PP1 is a ubiquitous phosphatase involved in many cellular processes, regulating metabolism, gene expression and RNA maturation, protein synthesis, cell cycle progression and stress responses. PP1 substrate specificity is provided by more than 200 PP1-interacting proteins (PIPs), or regulatory subunits. PIPs interact with PP1 catalytic subunit (PP1c) via a range of docking motifs to create holoenzymes that define subcellular localization and substrate specificity. Most PIPs (∼90%) contain an R/KVxF motif, allowing them to dock to a surface groove of PP1c without altering its conformation. Each PIP can thus be regarded as forming a ser/thr phosphatase with its own substrate specificity^29^,^34^,^35^,^36^. The KVTF motif identified from residues 117 to 120 in the C-terminal region of Pi04314 was mutated, resulting in a stable Pi04314mut protein that was unable to interact with PP1c isoforms, failing to re-localize them from the plant nucleolus (Fig. 5; Supplementary Fig. 9), indicating that the effector mimics regulatory subunits in its interaction with PP1c. Moreover, Pi04314mut also failed to enhance *P. infestans* leaf colonization, indicating that this activity requires interaction with PP1c.

There are three PP1c isoforms, α, β and γ (ref. 36), in mammals, and nine TOPP PP1c isoforms in *Arabidopsis*^37^ (Supplementary Fig. 2). Pi04314 interacts with three potato PP1c isoforms, PP1c-1, PP1c-2 and PP1c-3, which are related to TOPPs 1, 2, 4 and 5 (Supplementary Fig. 2). Indeed, no interaction in yeast or *in planta* was observed with a fourth potato isoform, PP1c-4 (Supplementary Fig. 11), which is similar to TOPPs 8 and 9 (Supplementary Fig. 2), indicating that Pi04314 is likely specific in its interactions to the three PP1c isoforms recovered from the Y2H screen. Many verified PIP regulatory subunits in mammalian systems also demonstrate interactions to specific PP1c isoform(s)^36^. Remarkably, silencing of *NbPP1c-1*, *NbPP1c-2* and *NbPP1c-3* did not significantly alter plant growth and development, although transcript levels were significantly reduced rather than abolished (Supplementary Fig. 10). However, as no silencing was observed of *NbPP1c-4*, it is also likely that plant PP1c isoforms share some functional redundancy, and that reduced levels of PP1c-1, PP1c-2 and PP1c-3 were compensated for by other isoforms.

Three lines of evidence indicate that Pi04314 likely forms a holoenzyme with each of the PP1c isoforms with which it interacts. First, silencing of *NbPP1c* isoforms attenuated *P. infestans* infection (Fig. 6), suggesting that their activities are important for late blight disease. As we did not silence *NbPP1c-4*, encoding an isoform that fails to interact with Pi04314 (Supplementary Fig. 11), the requirement for PP1c activity is thus associated with isoforms that interact with the effector. Second, when GFP–Pi04314 and GFP–Pi04314mut were each co-expressed with cMyc–PP1c-1 and immunoprecipitated using GFP–Trap_M beads, phosphatase activity was detected only with the PP1c-interacting WT form of the effector (Fig. 7). This implies that Pi04314 does not act as a phosphatase inhibitor. Third, transient expression of a phosphatase-dead version of just one isoform, PP1c–1mut, retained interaction with Pi04314 and acted as dominant negative to attenuate the enhanced infection promoted by *in planta* expression of Pi04314, and reduce leaf colonization in the absence of the effector (Fig. 8). Our data thus indicate that Pi04314 acts as a PIP to form a holoenzyme with specific PP1c isoforms, presumably to dephosphorylate key substrates in the plant cell.

Interestingly, the phosphatase-dead mRFP–PP1c–1mut no longer accumulated in the nucleolus (Fig. 8). Nevertheless, upon co-expression *in planta* with GFP–Pi04314 it significantly reduced the accumulation of the effector in the nucleolus. One interpretation of our observations is that the nucleolus contains a pool of active PP1c isoforms. Nucleolar sequestration of proteins has been observed previously, and was noted in the case of the mediator subunit Med19a, a target of *H. arabidopsidis* effector HpaRxLR44 (ref. 18). Further investigation, beyond the scope of the present study, is required to understand nucleolar accumulation of PP1c. However, the observation that PP1c–1mut retains interaction with Pi04314 *in planta* and reduces its presence in the nucleolus can be explained if this interaction occurs in the nucleoplasm, reducing the levels of Pi04314 entering the nucleolus to associate with active PP1c.

PIP regulatory substrates can re-localize PP1c isoforms to specific subcellular localizations, either to directly target specific substrates or to focus PP1c concentration at a site where multiple targets for dephosphorylation reside^35^,^36^. From the observation that focusing of Pi04314 in the host nucleus using an NLS did not reduce effector-mediated enhancement of *P. infestans* leaf colonization, coupled with the demonstration that Pi04314 re-localizes PP1c out of the nucleolus, we deduce that the substrates targeted for dephosphorylation by the Pi04314–PP1c holoenzyme reside in the nucleoplasm. Critically, we show that the pathogen itself re-localizes PP1c-1 from the nucleolus during the biotrophic phase of infection (Fig. 4), indicating that this is synonymous with disease development. Future efforts will focus on the identification and functional characterization of PP1c–Pi04314 substrates to better understand the molecular mechanisms by which this effector alters host processes.

The term susceptibility (S) factor has been coined to describe host proteins with detrimental effects on pathogen infection when mutated or silenced, and/or positive effects when overexpressed. S factors cover a range of host activities that support pathogen infection, from cellular alterations, to enhanced nutrition supporting pathogen growth, to suppression or antagonism of immunity^38^. There are few examples where the contribution of a host gene or protein to susceptibility is consequent upon direct effector activity. Examples include: the *P. syringae* type III effector AvrB, which mediates phosphorylation and activation of MAPK4, a suppressor of PTI^39^; and Xanthomonas transcription activator-like (TAL) effectors that directly upregulate *SWEET* genes, which contribute to sugar efflux and thus pathogen nutrition^40^. Silencing Pi04314-interacting isoforms of PP1c attenuated *P. infestans* infection, demonstrating that these effector targets fulfil the definition of S factors. However, based on expression of the effector *in planta*, it appears that their roles as S factors require R/KVxF-dependent interaction with Pi04314, with Pi04314 acting as a PIP to form unique ‘host–pathogen' holoenzymes. Phosphorylation plays many roles in immunity and, in the nucleus, is required for activation of transcription factors and regulating hormone pathways^11^,^12^. Indeed, the major JA regulator MYC2 (ref. 41), and the major SA regulator NPR1 (ref. 42), are activated by phosphorylation. It is thus interesting that transgenic potato lines expressing Pi04314 are attenuated in both JA and SA transcriptional responses (Fig. 9). In conclusion, it is, perhaps, unsurprising that a pathogen would employ dephosphorylation in the host nucleus to promote infection. However, effector Pi04314 acting as a regulatory subunit to co-opt host PP1c activity to the benefit of *P. infestans* is a remarkable example of molecular mimicry, and it will be fascinating to reveal the substrate(s) that are dephosphorylated by these holoenzymes to promote late blight disease.

## Methods

### Plasmid constructs

Full-length *StPP1c* genes were cloned from potato cDNA with gene-specific primers modified to contain the Gateway (Invitrogen) attB recombination sites. PCR products were purified and recombined into pDONR201 (Invitrogen) to generate entry clones via BP reactions using Gateway technology (Invitrogen). *Pi04314* and *SFI3*, minus signal peptide-encoding portions, were cloned into pDONR201 in the same way from *P. infestans* cDNA. Primer sequences are shown in Supplementary Table S1. Protein fusions were made by recombining the entry clones with the following plant expression vectors using LR clonase (Invitrogen). N-terminal GFP, mRFP and cMyc fusions were made by recombining the entry clones with pB7WGF2, pK7WGR2 and pGWB18, respectively. The mis-targeted form of the GFP–effector fusion _myr_GFP–Pi04314 was created using the same method as described for PITG_04097 (ref. 19). The _NLS_GFP–Pi04314 construct was made by creating a modified form of pB7WGF2 with an NLS signal derived from SV40 T antigen (amino acid sequence PKKKRKV^28^) added to the N terminus of the GFP. VIGS constructs were synthesized by GenScript. Briefly, ∼100 bp fragments of the non-conserved 5′ regions of each PP1c interactor were concatenated to make combined 5′ inserts and the same was performed with 3′ regions to make 3′ inserts. These were cloned into pBinary TRV vectors^43^ between *Hpa*I and *Eco*RI sites in the antisense orientation. A TRV construct expressing GFP described previously was used as a control^26^. The two largest leaves of four leaf-stage *N. benthamiana* plants were pressure-infiltrated with LBA4404 *Agrobacterium tumefaciens* strains containing a mixture of RNA1 and each PP1c VIGS construct or the GFP control at OD_600_=0.5. Plants were used for assays or to check gene silencing levels by qRT–PCR 3 weeks later.

### Plant material

*N. benthamiana* plants were grown under a 16-h day at 22 °C and an 8-h night at 18 °C. Supplementary lighting when the ambient light dropped below 200 W m^−2^, and shading when it was above 450 W m^−2^ were automatically provided. Potato plantlets were propagated on Murashige and Skoog (MS) medium in growth chambers (16/8-h light/dark cycle at 20 °C).Three-week-old plantlets were transplanted and grown in individual pots in a greenhouse at 20–26 °C for the further assays.

### Potato transformation

*Agrobacterium* containing the overexpression vector PRI101–Pi04314 was transformed into the potato cultivar E3 by microtuber disc transformation according to Si *et al*.^44^ and Tian *et al*.^45^. Positive lines, which were first screened on differential medium (3% MS+0.2 mg l^−1^ indole-3-acetic acid (IAA)+0.2 mg l^−1^ gibberellic acid (GA3)+0.5 mg l^−1^ 6-benzylaminopurine (6-BA)+2 mg l^−1^ zeatin (ZT)+75 mg l^−1^ kanamycin (Kan)+200 mg l^−1^ Cefotaxime (Cef), pH 5.9), and then transferred to root generation medium (3% MS+50 mg l^−1^ Kan+400 mg l^−1^ Cef, pH 5.9), were then confirmed by the PCR with the forward primer of 35S promotor and gene-specific reverse primer of Pi04314. Gene expression levels of Pi04314 were analysed by semi-quantified PCR (the primers are shown in the Supplementary Table 1).

### Agroinfiltration and infection assays

*A. tumefaciens* strain AGL1 containing plasmid constructs were grown overnight in yeast-extract and beef (YEB) medium with appropriate antibiotics at 28 °C. The bacteria were pelleted, resuspended in infiltration buffer (10 mM 2-(N-morpholino)ethanesulfonic acid (MES), 10 mM MgCl_2_ and 200 μM acetosyringone) and adjusted to the required OD_600_ before infiltration into *N. benthamiana* leaves (the OD_600_ was generally 0.005–0.01 for imaging purposes and 0.5 for immunoblots, immunoprecipitation and activity assays). For co-expression of multiple constructs agrobacterial suspensions carrying the different constructs were thoroughly mixed before infiltration.

*P. infestans* strain 88069 was cultured on rye agar at 19 °C for 2 weeks before collecting the inoculum. The plates were flooded with 5 ml H_2_O, and scraped to release sporangia. The sporangial suspension was poured into a Falcon tube, and sporangia numbers were counted using a haemocytometer then adjusted to 30,000 sporangia per ml; 10 μl droplets were inoculated onto the abaxial side of detached *N. benthamiana* leaves stored on moist tissue in sealed boxes. For VIGS, the number of inoculated lesions sporulating at 7 d.p.i. were counted and expressed as a percentage increase in sporulating lesions compared with the GFP control plants. Sporangia counts were performed on 10 d.p.i. leaves from VIGSed plants, which had been immersed in 5 ml H_2_O and vortexed to release sporangia. A haemocytometer was used to count the number of sporangia recovered from each leaf and was expressed as sporangia per ml. *A. tumefaciens* transient expression in combination with *P. infestans* infection were carried out as described previously^26^. Briefly, *Agrobacterium* suspensions at concentrations of OD_600_=0.1 infiltrated into leaves and after 1 day, each infiltration site was inoculated with 10 μl of *P. infestans* inoculum at 30,000 sporangia per ml. Lesion sizes were measured at 7 d.p.i. For potato pathology tests, *P. infestans* isolate HB09-14-2 (race 1.2.3.4.5.6.7.8.9.10.11), collected from Hubei Province, China, and was cultured on rye and sucrose agar (RSA) medium at 18 °C for 13 days for potato infection as described by He *et al*.^46^. Sporangia were washed with ddH_2_O to a concentration of 7 × 10^4^ sporangia per ml. Disease development was recorded by detached leaf assays as previously described^46^. Twelve leaves from six individual plants were used for each of three replicates. All the data were analysed by ANOVA.

### Live cell imaging

*N. benthamiana* leaf cells were imaged no later than 2 days after agroinfiltration using Leica TCS SP2 AOBS, Ziess 710 or Nikon A1R confocal microscopes with the following water-dipping objectives: Leica HCX PL APO lbd.BL × 63/1.20 W and L × 40/0.8, Zeiss PL APO × 40/1.0 or Nikon × 60/1.0 W. GFP was excited with 488 nm from an argon laser and its emissions were detected between 500 and 530 nm. mRFP was excited with 561 nm from a diode laser, and its emissions were collected between 600 and 630 nm. The pinhole was set at 1 airy unit for the longest wavelength of light being used. Images were only collected from leaf cells expressing low levels of the protein fusions to minimize possible artefacts of ectopic protein expression. Cells for imaging and quantification to measure the effects of co-expression of effectors and the PP1c isoforms were selected that had generally low levels of expression as with all of the imaging but for these assays particularly cells that had relative levels of effector expression sufficient to have an impact on the co-expressed PP1c. Images were projected, processed and quantified using the ImageJ, Leica LCS, Zen 2010 or NIS-Elements software packages as required. The nucleolar to nucleoplasmic ratio of fluorescence intensity was chosen as a measure of re-localization to account for variation of protein expression levels from cell to cell. Single optical sections that captured the brightest section through the nucleolus were collected, and the mean intensities of fluorescence were measured in the nucleoplasm and nucleolus separately after drawing regions of interest to encompass them. Where the optical section was collected at the point at which nucleolar labelling was only present in the ring at the edge of the nucleolus, the mean fluorescence intensity of the ring was measured using a suitable region of interest polygon. Images were processed with Adobe Photoshop CS2 and Adobe Illustrator for figures.

### Immunoprecipitation and phosphatase assay

N-terminal GFP, mRFP and cMyc-tagged phosphatase PP1C, and Pi04314 or SFI3 were overexpressed in *N. benthamiana* using *Agrobacterium*-mediated expression. Leaf samples were collected on the second day of infection and proteins were extracted using GTEN (10% (vol/vol) glycerol, 25mM Tris-HCl (pH 7.5), 1 mM EDTA, 150 mM NaCl) buffer with 10 mM DTT, protease inhibitor cocktail, 1 mM phenylmethyl sulphonyl fluoride and 0.2% Nonidet P-40. To immunoprecipitate GFP-tagged PP1C, protein extracts were incubated with GFP–Trap_M beads (Chromtek) for 1 h at 4 °C. Beads were washed three times in GTEN buffer containing protease inhibitor cocktail and 1 mM phenylmethyl sulphonyl fluoride, and were either resuspended in wash buffer for the phosphatase assay or were mixed with 2 × SDS–polyacrylamide gel electrophoresis sample buffer and analysed by immunoblotting. Samples were loaded onto a 4–12% Bis-Tris NuPAGE Novex gel run with 1 × MES SDS running buffer for 1 h at 180 V (Invitrogen). Gels were blotted onto a nitrocellulose membrane for 1.5 h at 30 V and stained with ponceau solution to show loading and transfer. Membranes were blocked in 4% milk in 1 × PBST (phosphate buffered saline (137 mM NaCl, 12 mM Phosphate, 2.7 mM KCl, pH 7.4) with Tween-20 0.2% (vol/vol)) (0.2% Tween 20) before addition of the primary antibodies at 1:2,000 dilutions: either a monoclonal GFP antibody raised in mouse (Sigma-Aldrich, cat. no. G1546), a monoclonal anti cMyc antibody raised in mouse (Santa Cruz, cat. no. SC-40) or a polyclonal anti mRFP antibody raised in rabbit (Sigma-Aldrich, cat. no. 5F8). The membrane was washed with 1 × PBST (0.2% Tween 20) before addition of the secondary antibody at 1:8,000 dilution; either anti-mouse Ig-HRP antibody (Sigma-Aldrich, cat. no. A9044) or anti-rabbit Ig-HRP antibody (Sigma-Aldrich, cat. no. Ab6836). ECL (Amersham) detection was used according to the manufacturer's instructions. The original gel images are shown in Supplementary Fig. 15. The phosphatase activity assay was performed directly on the resuspended beads by incubating with phospho substrate provided in the Ser/Thr phosphatase assay kit 1 (Millipore), and the activity was measured using malachite green-based assay using a plate-reading spectrophotometer according to the manufacturer's instructions.

### Yeast-2-hybrid

Y2H screening was performed using the ProQuest system (Invitrogen). DNA-binding domain ‘bait' fusions were generated by recombination between pDonr201–Pi04314 and pDEST32, generating pDest32–Pi04314. This construct was transformed into yeast strain MaV203, and nutritional selection used to recover transformants. A single transformant was grown up and used to prepare competent yeast cells, which then were transformed with a potato Y2H ‘prey' library, commercially prepared from *P. infestans* infected leaf material at 15 and 72 hours post infection (hpi)^16^. Interactions were confirmed using reporter gene assays namely ability to grow on media missing histidine (−HIS), and screening for gain of β-galactosidase activity (β-gal). Candidate interacting preys (pDEST22) were confirmed by retransformation with the Pi04314 bait construct or with a pDest32–SFI3 control to rule out the possibility that the observed reporter gene activation had resulted from interactions between the prey, and the DNA-binding domain of the bait construct or DNA-binding activity of the prey itself. StPP1c-4 was also cloned into the prey vector to obtain pDEST22–StPP1c-4, which was also tested pairwise for an interaction with pDEST32–Pi04314.

### Plant treatments

Six-week-old potato plants were used for the treatments. Three leaves from the third to the fifth compound leaf from the top of each potato line were infiltrated with flg22 (10 mM). Six detached leaves from each line were treated by spraying with exogenous 1 mM meJA (the solutions contained 10 μl ethanol per ml) or 1 mM SA (the solutions contained 10 μl dimethylsulphoxide per ml), separately. Treated leaves were kept in clean boxes under humid conditions. For each treatment, leaves were sprayed with a total of 20 ml of each hormone solution until runoff. Three leaves per time point each were collected from separate plants and snap-frozen in liquid nitrogen. Three biological replicates were independently tested for each treatment.

### Gene expression analysis

For gene expression analysis, RNA was extracted using an RNeasy kit with on column DNA digestion (Qiagen) to remove DNA contamination according to the manufacturer's instructions. First-strand cDNA was synthesized from 2 μg of RNA using SuperScript II RNase H reverse transcriptase (Invitrogen) according to manufacturer's instructions. Realtime qRT–PCR reactions were performed using Power SYBR Green (Applied Biosystems), and run on a Chromo4 thermal cycler (MJ Research, UK) using Opticon Monitor 3 software. Primer pairs were designed outside the region of cDNA targeted for silencing following the manufacturer's guidelines. Primer sequences are shown in Supplementary Table 1. Relative expression of the target genes was calculated using the 2^−ΔΔC^T method^47^ with the *StUBI* housekeeping gene as the reference for potato, *NbEf1a* for *N. benthamiana* and *PiActA* for *P. infestans*. In each case, PCR conditions were 95 °C for 15 min, followed by 40 cycles of 95 °C for 15 s, 60 °C for 30 s and 72 °C for 30 s. Results from three biological replicates were tested independently, and expression in transgenic lines OE-6 and OE-8 was compared with that in the control E3 line using one-way ANOVA. For analyses of meJA and SA responses, the following potato genes were selected from recent microarray data^33^: StJAZ1-like (also referred to as StJas: transcript number PGSC0003DMT400007592), and StMYC2-like (PGSC0003DMT400031899) for meJA; and StWRKY40-like (also referred to as StmRNA; PGSC0003DMT400019061), and StWRKY16-like (similar to WRKY50 from tomato; PGSC0003DMT400080127) for SA. Microarray profiles for these genes following hormone treatments are found by entering the transcript number on the searchable database (https://ics.hutton.ac.uk/solarray/) and clicking the probe number.

## Additional information

**How to cite this article:** Boevink, P. C. *et al*. A *Phytophthora infestans* RXLR effector targets plant PP1c isoforms that promote late blight disease. *Nat. Commun*. 7:10311 doi: 10.1038/ncomms10311 (2016).

## Supplementary Material

Supplementary Information Supplementary Figures 1-15 and Supplementary Table 1
