# Supplementary material for: A Phytophthora infestans RXLR effector targets plant PP1c isoforms that promote late blight disease
Source: Nat Commun. 2016 Jan 29;7:10311. doi: 10.1038/ncomms10311 (PMC4740116; doi:10.1038/ncomms10311)
Supplement: Supplementary Information — Supplementary Figures 1-15 and Supplementary Table 1 [file ncomms10311-s1.pdf]

Supplementary Data

Supplementary Figures

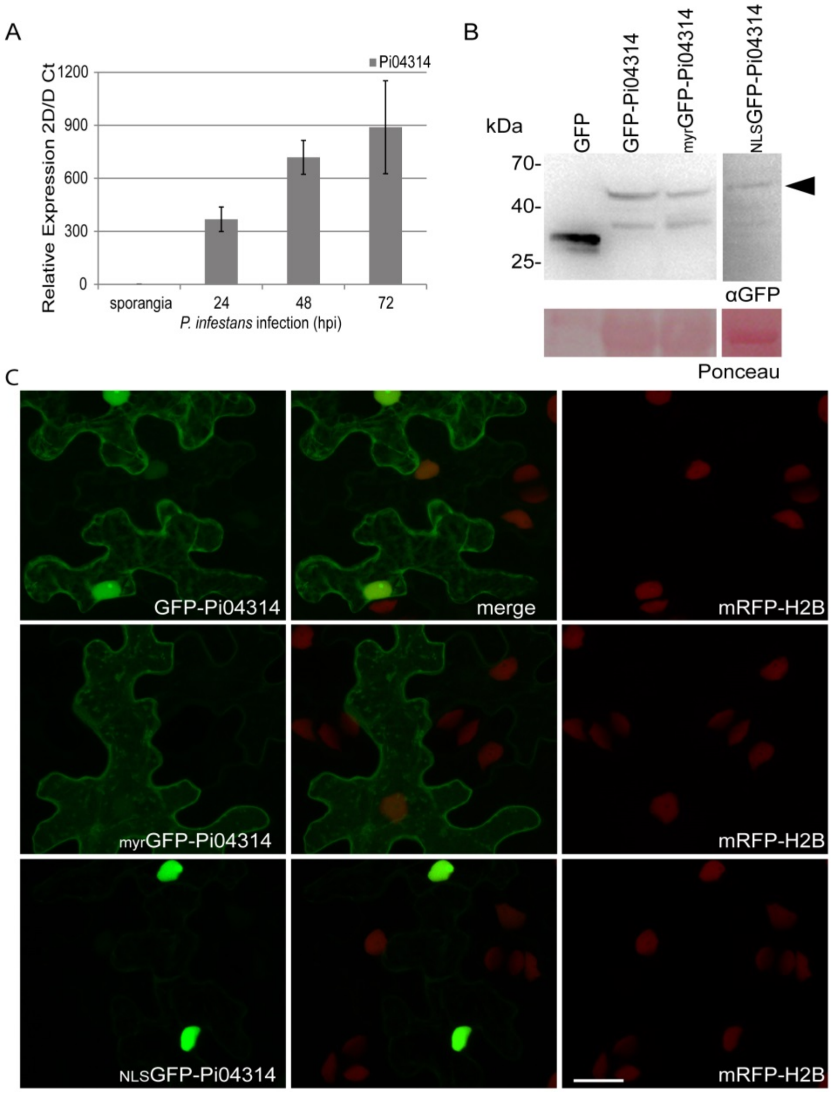

Supplementary Figure 1. Pi04314 is expressed during infection, each GFP-Pi04314 fusion is stable and $_{myr}$GFP-Pi04314 is removed from the nucleus while $_{NLS}$GFP-Pi04314 is concentrated in the nucleus.

(A) Graph of qRT-PCR results indicating that the effector Pi04314 is specifically upregulated during early infection stages between 24 and 72 hours after inoculation compared to sporangia.

(B) Western blotting indicates that the GFP-04314 fusion and the two modified forms of the fusion, with myristoylation (myr) or nuclear localisation (NLS) signals, are all similarly stable. Arrowhead indicates the full-size fusion proteins. Protein loading is indicated by ponceau stain and the antibody used is αGFP.

(C) Images are projections of confocal Z series of the three fusions expressed in a transgenic *N. benthamiana* line expressing an mRFP tagged histone (mRFP-H2B) to mark the nucleus. GFP-Pi04314 is localised to the nucleus with cytoplasmic background, $_{myr}$GFP-Pi04314 is excluded from the nucleus and $_{NLS}$GFP-Pi04314 is focused in the nucleus. Scale bar is 20 μm.

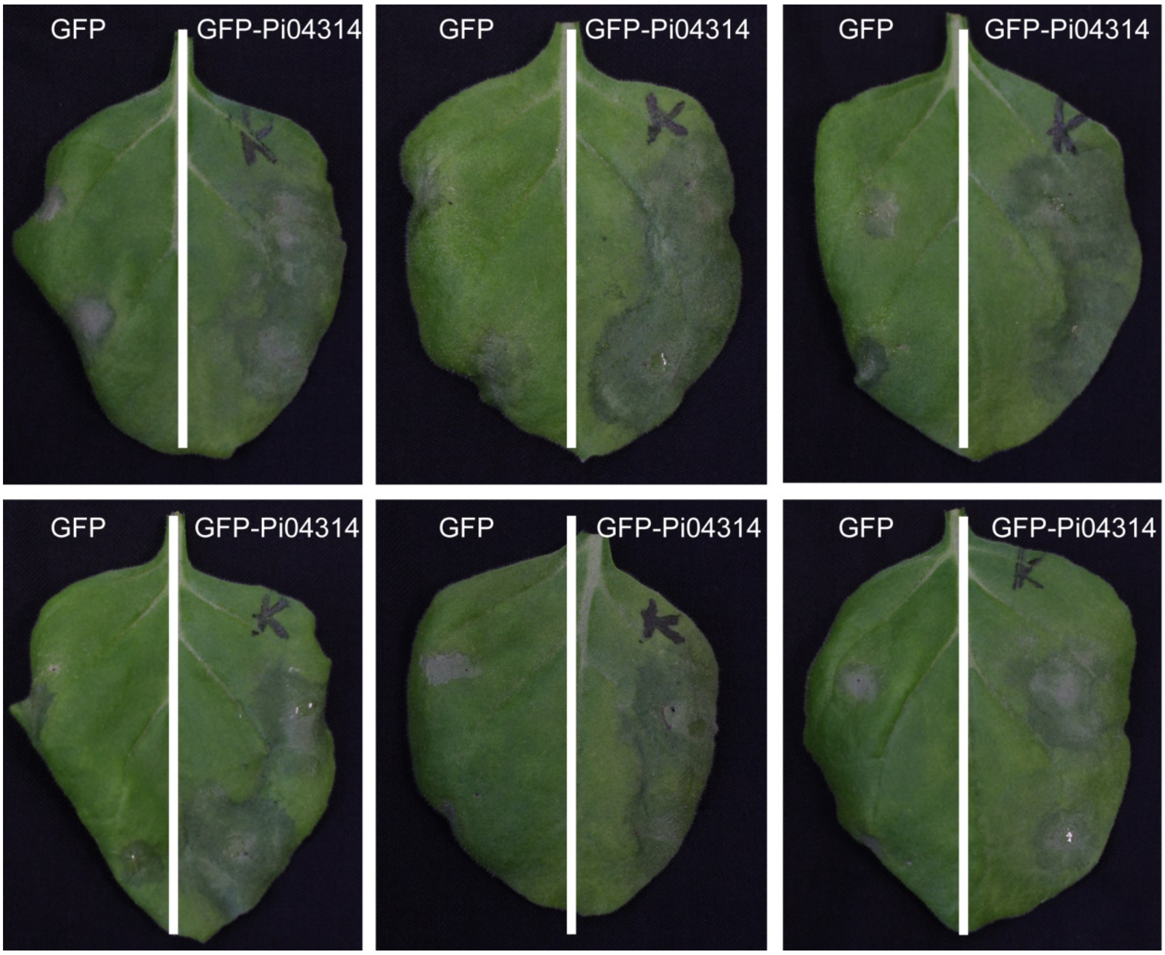

Supplementary Figure 2. GFP-Pi04314 promotes *P. infestans* infection. Example leaves showing the increased growth of *P. infestans* on the side of each leaf infiltrated with the GFP-Pi04314 compared to the side infiltrated with the GFP control.

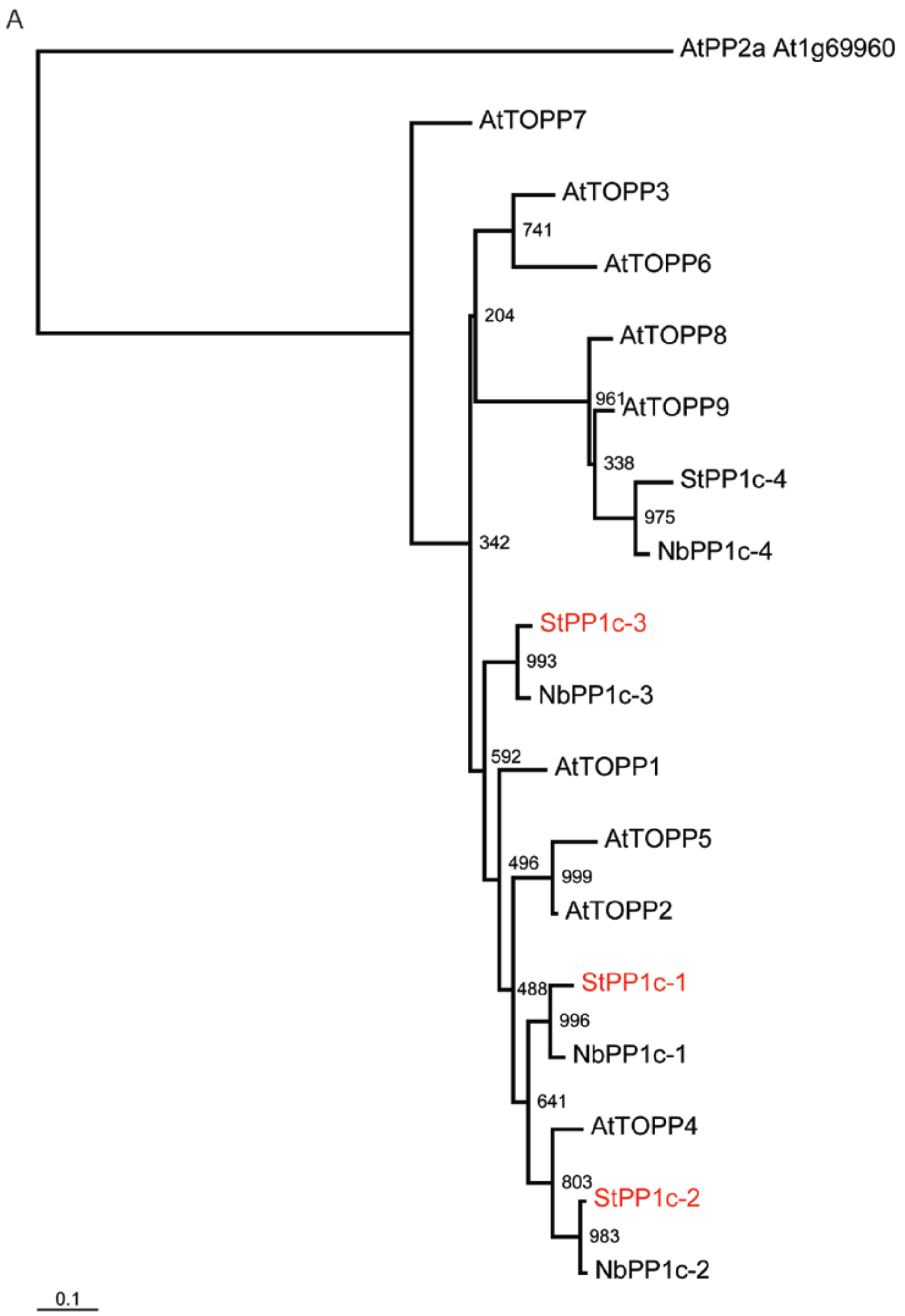
A
AtPP2a At1g69960
AtTOPP7
AtTOPP3
741
AtTOPP6
204
AtTOPP8
961
AtTOPP9
338
StPP1c-4
975
NbPP1c-4
342
StPP1c-3
993
NbPP1c-3
592
AtTOPP1
AtTOPP5
496
999
AtTOPP2
488
StPP1c-1
996
NbPP1c-1
641
AtTOPP4
803
StPP1c-2
983
NbPP1c-2
0.1

B

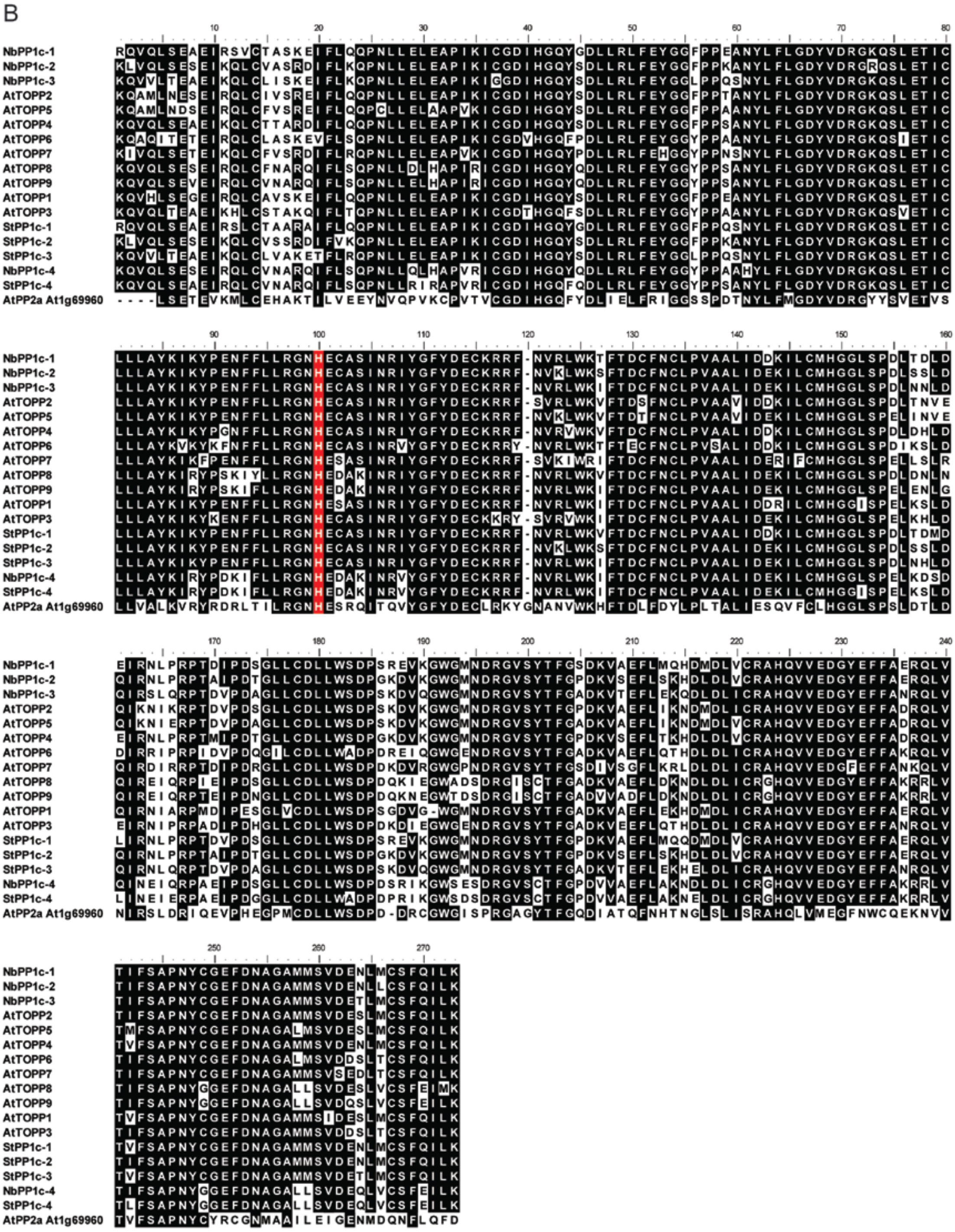
NbPP1c-1
NbPP1c-2
NbPP1c-3
AtTOPP2
AtTOPP5
AtTOPP4
AtTOPP6
AtTOPP7
AtTOPP8
AtTOPP9
AtTOPP1
AtTOPP3
StPP1c-1
StPP1c-2
StPP1c-3
NbPP1c-4
StPP1c-4
AtPP2a At1g69960

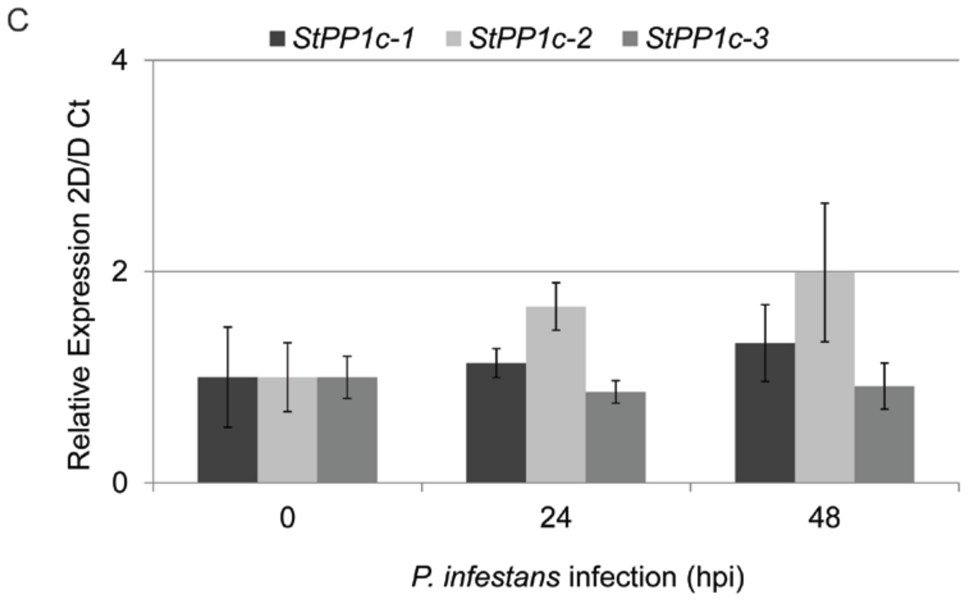

Supplementary Figure 3. Phylogenetic tree and alignment of Arabidopsis, *N. benthamiana* and *S. tuberosum* PP1c sequences

(A) Phylogenetic tree of PP1c sequences from Arabidopsis, *N. benthamiana* and *S. tuberosum* rooted with an Arabidopsis PP2a sequence shows the potato PP1c isoforms targeted by effector Pi04314 cluster separately from the non-interacting PP1c-4. Potato PP1c-1 and PP1c-2 cluster with the Arabidopsis TOPP4. A maximum likelihood tree was produced for the alignment using TOPALi v2.5 with 1000 bootstraps. The resulting tree was rendered with TreeView. The sequences in red are the interacting clones identified in the Y2H screen.

(B) The amino acid alignment used to generate the tree was performed by CLUSTALW and trimmed to remove poorly conserved regions. The residue highlighted in red is the amino acid mutated in StPP1c-1 to make the His129Ala mutant PP1c-1mut.

(C) QRT-PCR analysis of *StPP1c-1, StPP1c-2* and *StPP1c-3* at 24 and 48 hours post-inoculation of *P. infestans* on potato cv Desiree, relative to expression in uninfected potato leaves (0), which was given a value of 1.

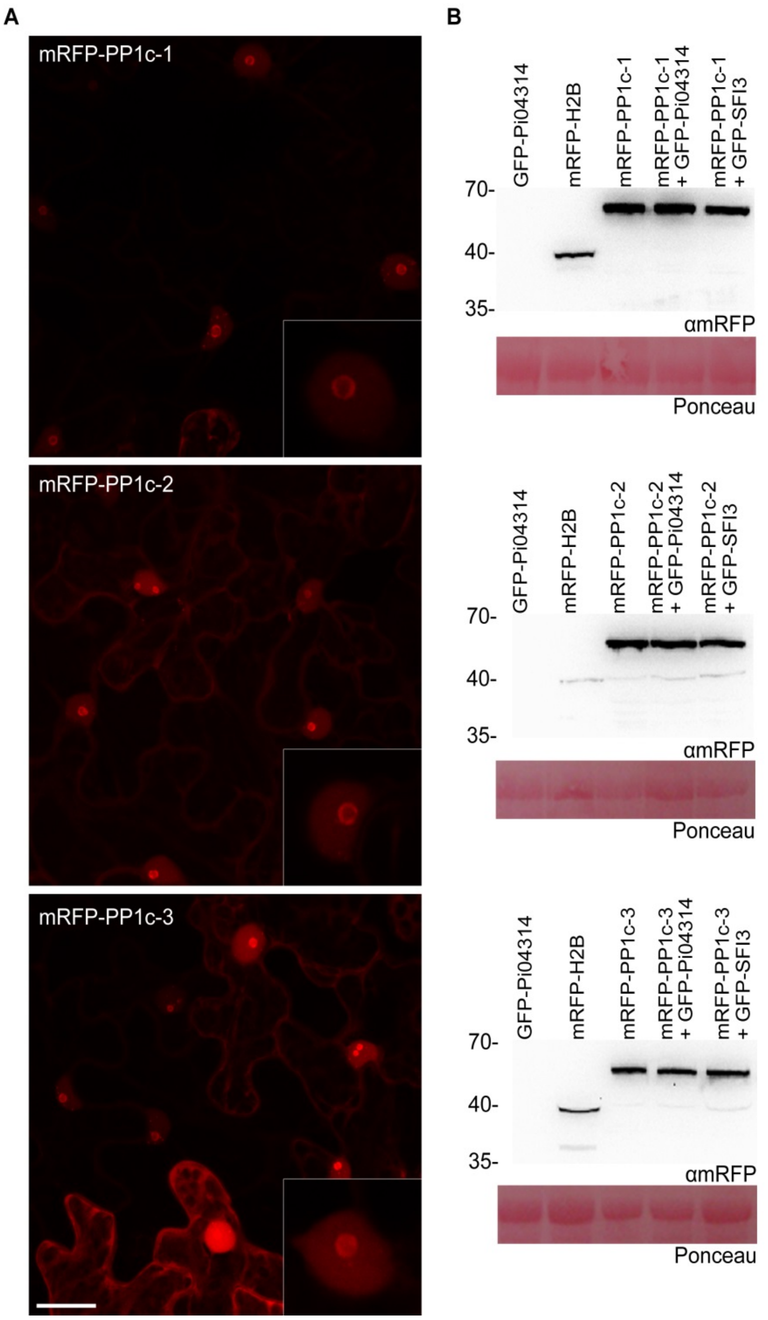

Supplementary Figure 4. mRFP-PP1c fusions are localised to the nucleus and nucleolus and are equally stable alone or in the presence of Pi04314 and SFI3.

(A) Images are projections of confocal z series of each of the interacting PP1c isoform expressed alone as mRFP fusions which accumulate in the nucleolus. Insets are single optical sections through one of the nuclei from each image. Scale bar is 20 μm.

(B) Western blots probed with mRFP antibody (αmRFP) show that the mRFP-PP1c fusions are stable alone and when co-expressed with GFP-Pi04314 and GFP-SFI3. Size markers are in kDa. Protein loading is indicated by the Ponceau stain.

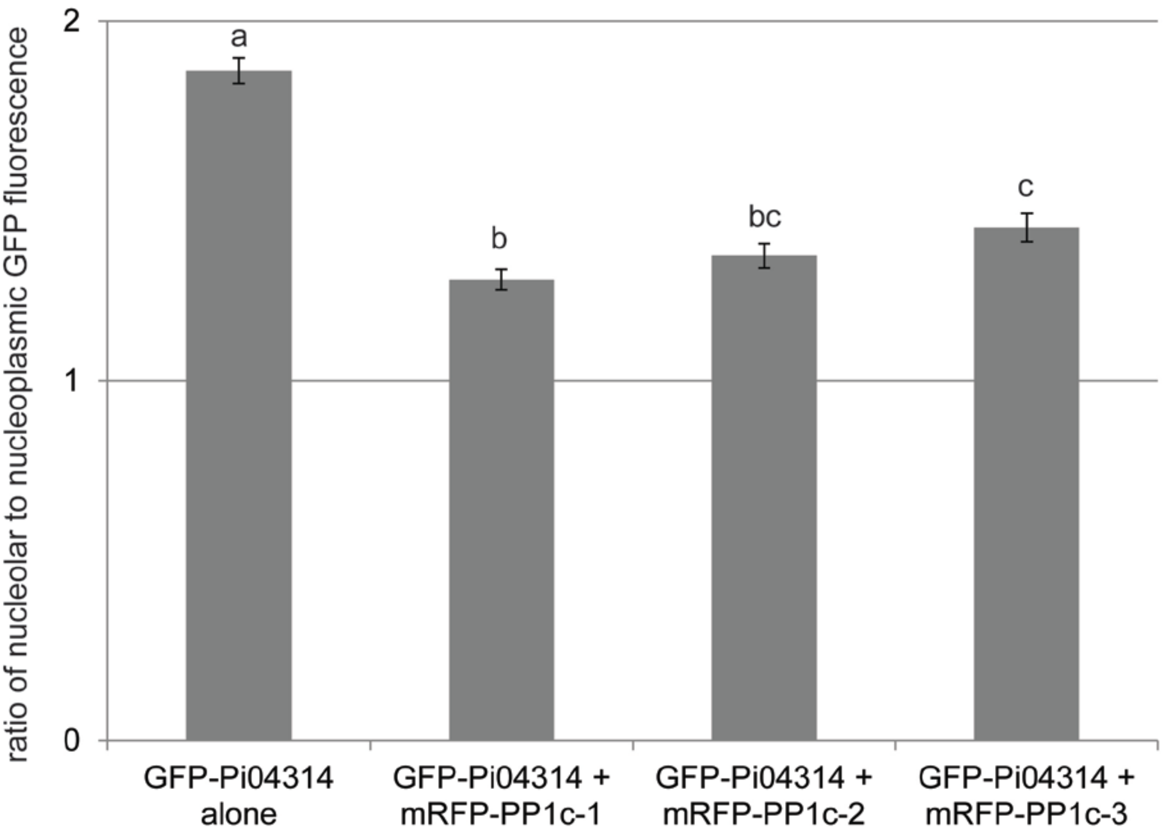

Supplementary Figure 5. GFP-Pi04314 is also relocalised out of the nucleolus on co-expression with PP1c fusions.

Graph showing that the GFP-Pi04314 accumulation in the nucleolus is reduced when co-expressed with mRFP-PP1c isoform fusions. Error bars are standard error and the graph represents the combined data from 3 biological replicates (n = 35 per construct), letters on graph denote statistically significant differences (ANOVA p>0.001 [except b to c where p=0.045]).

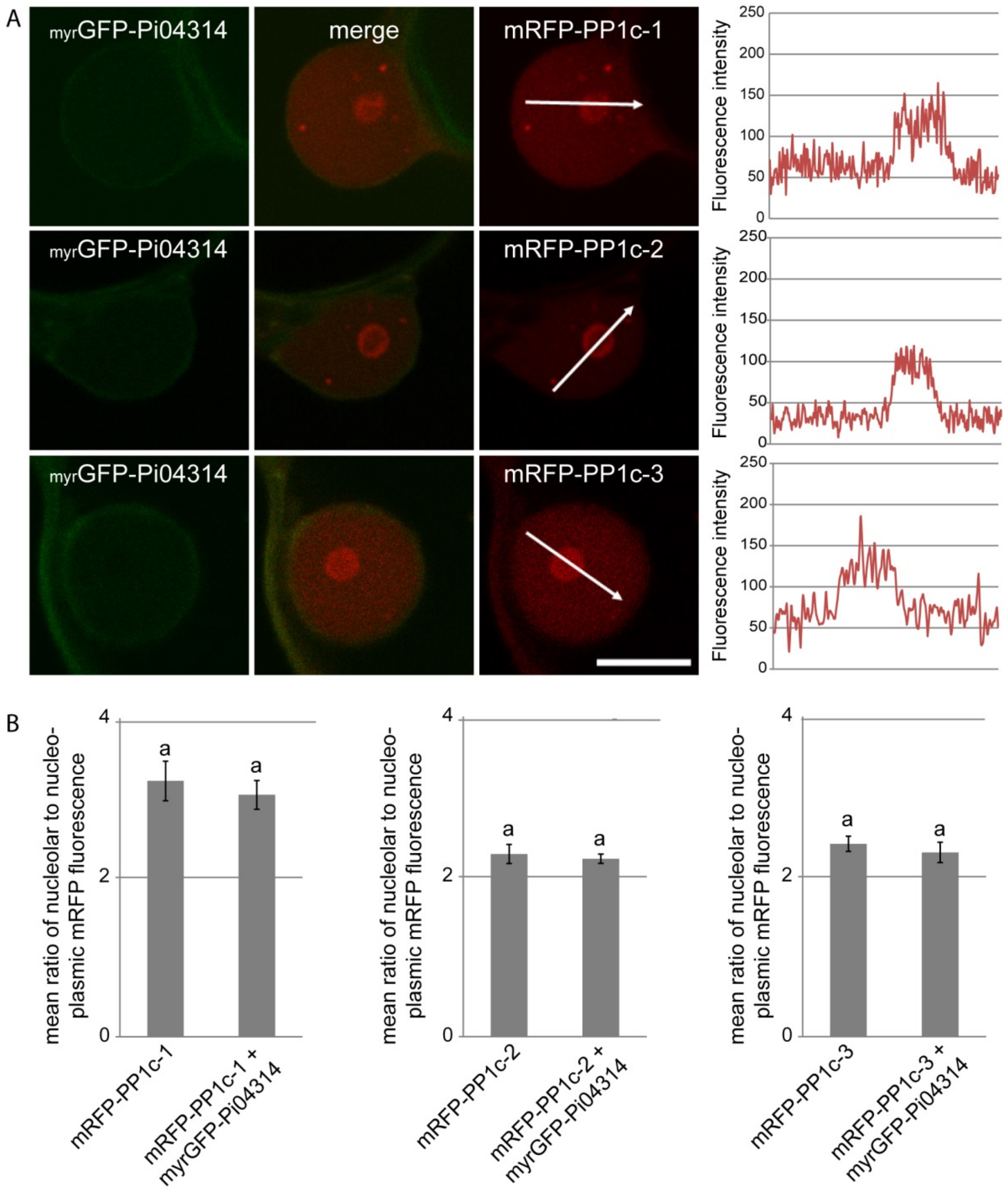

Supplementary Figure 6. $_{myr}$GFP-Pi04314 does not cause a loss of mRFP-PP1c from the nucleolus.

(A) Images are single optical sections through example nuclei co-expressing $_{myr}$GFP-Pi04314 and the mRFP-PP1c isoforms, showing that the mRFP-PP1c isoforms are not relocalised by $_{myr}$GFP-Pi04314. Scale bar is 10 μm. White arrows indicate mRFP fluorescence intensity plots shown in graphs to the right of each image.

(B) Graphs of the average ratios of nucleolar to nucleoplasmic mRFP fluorescence from the mRFP-PP1c fusions expressed alone or with $_{myr}$GFP-Pi04314 showing there was no significant reduction in co-expressing cells. Error bars are standard error and the graph represents the combined data from 3 biological reps (n = 40 per construct), letters on graph denote statistically insignificant differences (ANOVA $p>0.95$).

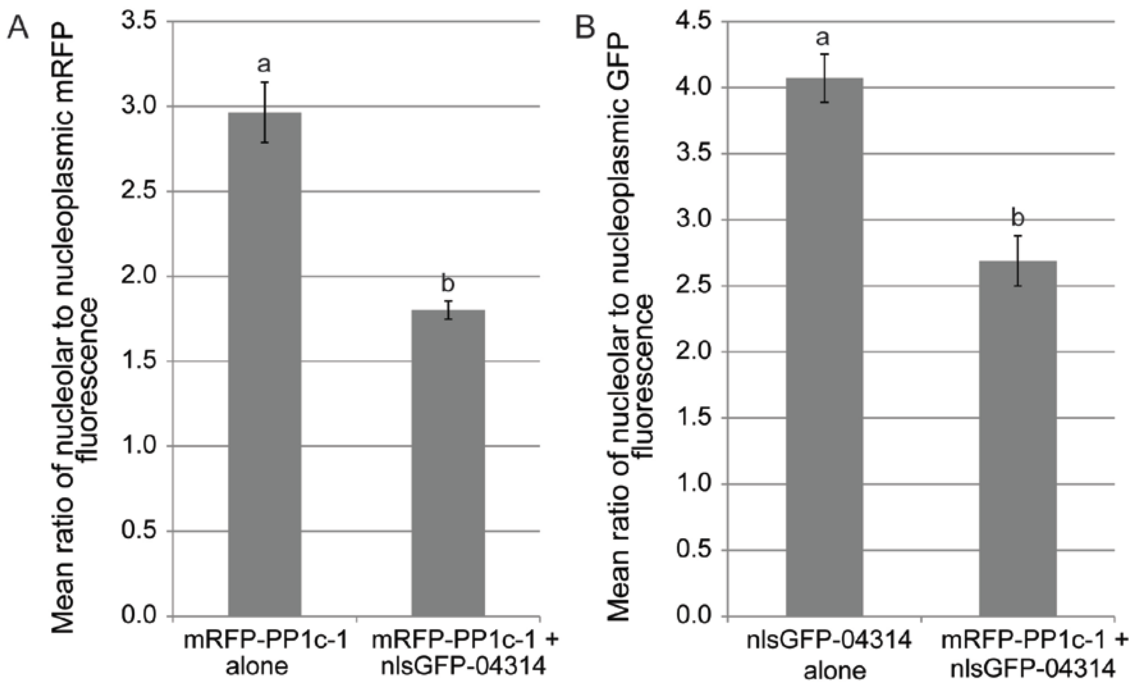

Supplementary Figure 7. Co-expression of $_{NLS}$GFP-Pi04314 with mRFP-PP1c-1 results in reduced GFP and mRFP fluorescence in the nucleolus.

(A) Graph of the average ratio of nucleolar to nucleoplasmic mRFP fluorescence from the mRFP-PP1c-1 fusion expressed alone or with $_{NLS}$GFP-Pi04314 showing there was a significant reduction in co-expressing cells. Error bars are standard error and the graph represents the combined data from 3 biological reps (n = 40 per construct), letters on graph denote statistically significant differences (ANOVA p<0.001).

(B) Graph of the average ratio of nucleolar to nucleoplasmic GFP fluorescence from the $_{NLS}$GFP-Pi04314 fusion expressed alone or with mRFP-PP1c-1 showing there was a significant reduction in co-expressing cells. Error bars are standard error and the graph represents the combined data from 3 biological reps (n = 40 per construct), letters on graph denote statistically significant differences (ANOVA p<0.001).

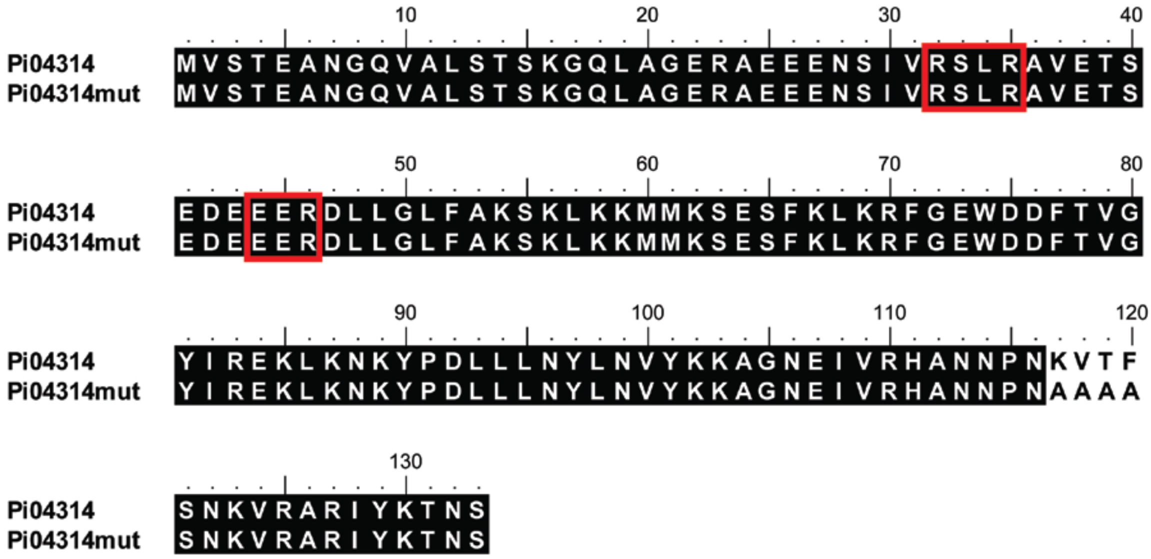

Supplementary Figure 8. Alignment of the protein sequences of the wild type and mutant forms of the effector Pi04314. (Related to Fig. 4).

The wild type and mutant Pi04314 amino acid sequences aligned. The conserved RXLR and EER motifs are boxed in red and the location of the R/KVxF PP1c binding motif (KVTF) and its mutation to alanines in Pi04314mut are shown with a white background.

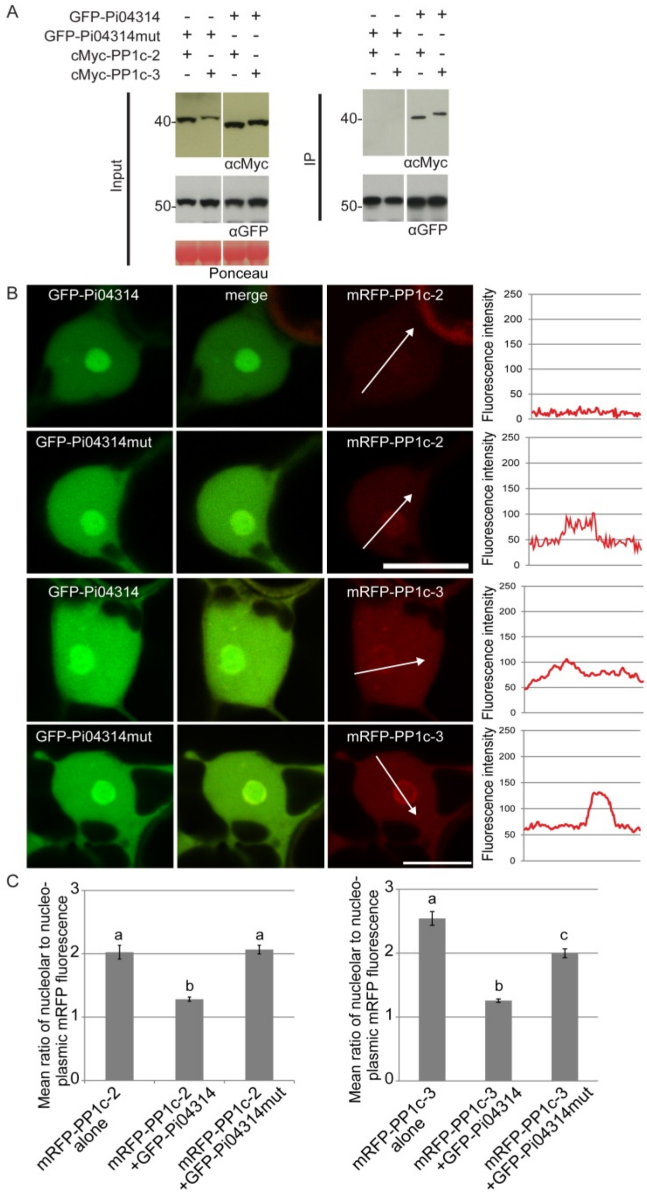

Supplementary Figure 9. The Pi04314 KVxF mutant does not interact with PP1c proteins.

(A) Immunoprecipitation of protein extracts from agroinfiltrated leaves using GFP-trap shows that PP1c-2 and -3 also lose their association with Pi04314mut. Expression of constructs in the leaves is indicated by +. Protein size markers are indicated in kDa, and protein loading is indicated by Ponceau stain.

(B) Representative single optical section images comparing the co-expression of the mRFP-PP1c isoforms 2 and 3 with GFP-Pi04314 (reduced nucleolar fluorescence) or GFP-Pi04314mut (normal nucleolar fluorescence). Scale bar is 10 μm. White arrows indicate fluorescence intensity plots in graphs to the right of each image.

(C) Graphs of the ratios of nucleolar to nucleoplasmic ratios indicate that GFP-Pi04314mut does not cause as much of a reduction in nucleolar labelling by the mRFP-PP1c isoforms 2 and 3 as the wild type GFP-Pi04314. Error bars are standard error and the graph represents the combined data from 3 biological reps (n = 40 per construct), letters on graph denote statistically significant differences (ANOVA p>0.001).

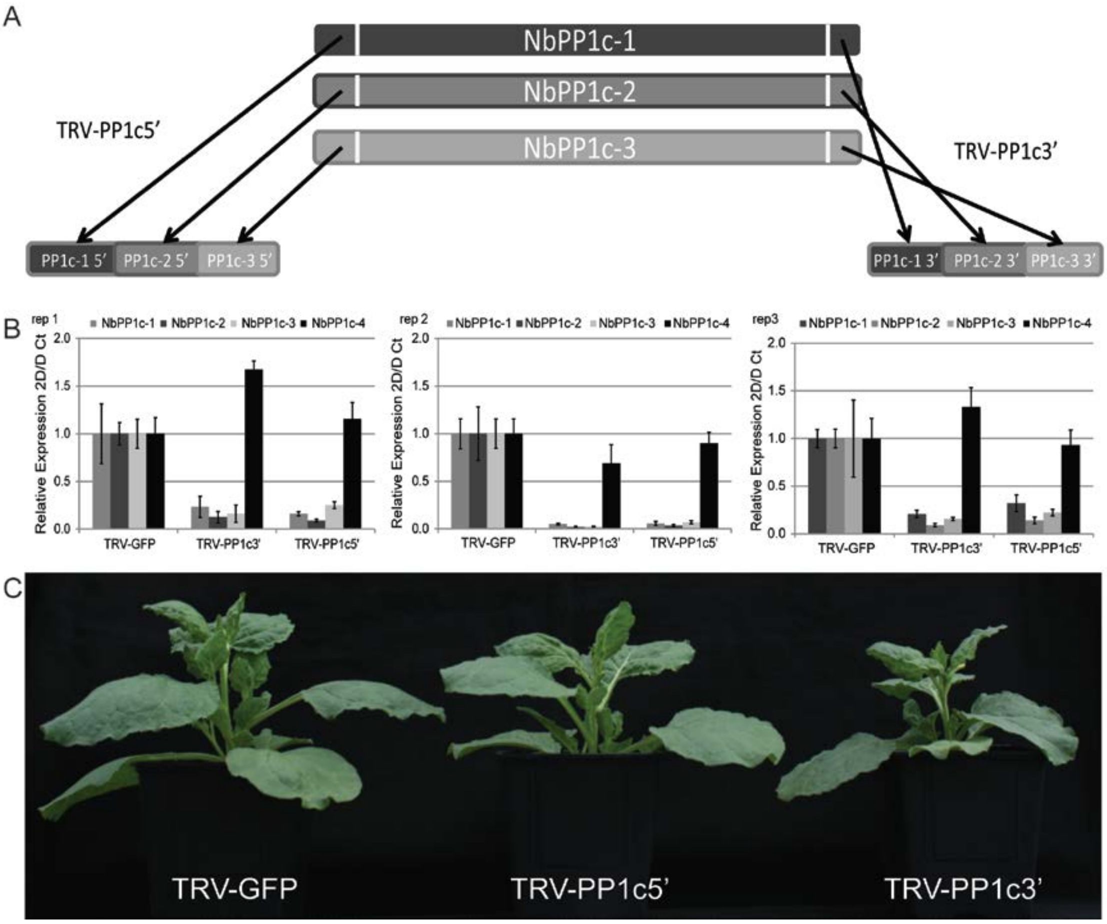

Supplementary Fig. 10. Silencing vector construction, silencing levels and plant phenotypes.

(A) Diagrammatic representation of the sequences of the targeted PP1c isoforms and the variable sequence regions at either end selected for inclusion in the silencing constructs.

(B) Analysis of the extent of silencing of the three targeted PP1c isoforms compared to the off-target silencing control PP1c-4 using qRT-PCR. The targeted genes exhibit 80-95% reduction in expression in TRV-PP1c5' & 3' plants compared to TRV-GFP whereas the off-target silencing control expression is comparable in each plant line.

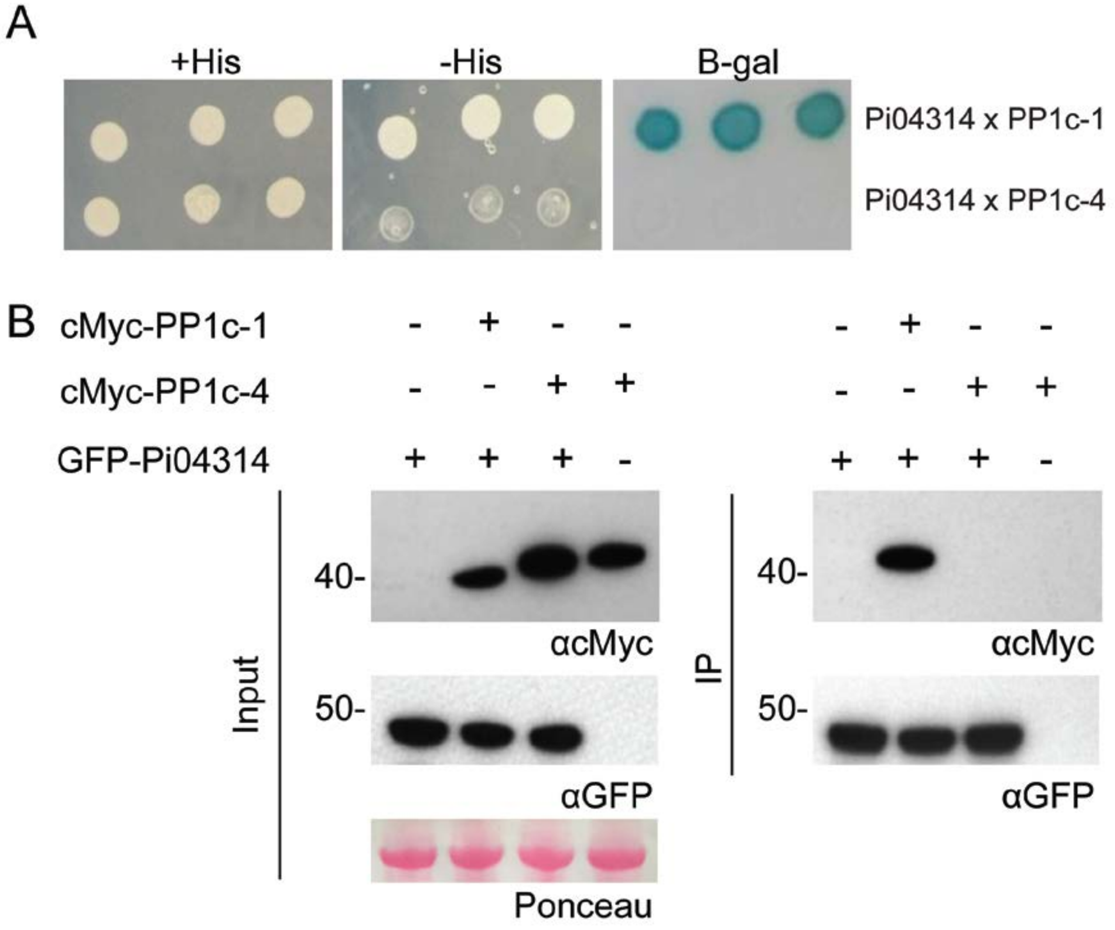

Supplementary Figure 11. The off-target silencing control PP1c-4 does not interact with Pi04314.

(A) Yeast co-expressing the PP1c-1 with Pi04314 grew on non-selective medium (+HIS) and selective medium (-HIS) and had β-galactosidase (B-Gal) activity while those co-expressing PP1c-4 did not.
(B) The lack of interaction between PP1c-4 and the effector was confirmed by immunoprecipitation using GFP trap, cMyc-PP1c-1 was immunoprecipitated with GFP-Pi04314 whereas cMyc-PP1c-4 was not. Expression of constructs in the leaves is indicated by +. Protein size markers are indicated in kDa, and protein loading is indicated by Ponceau stain.

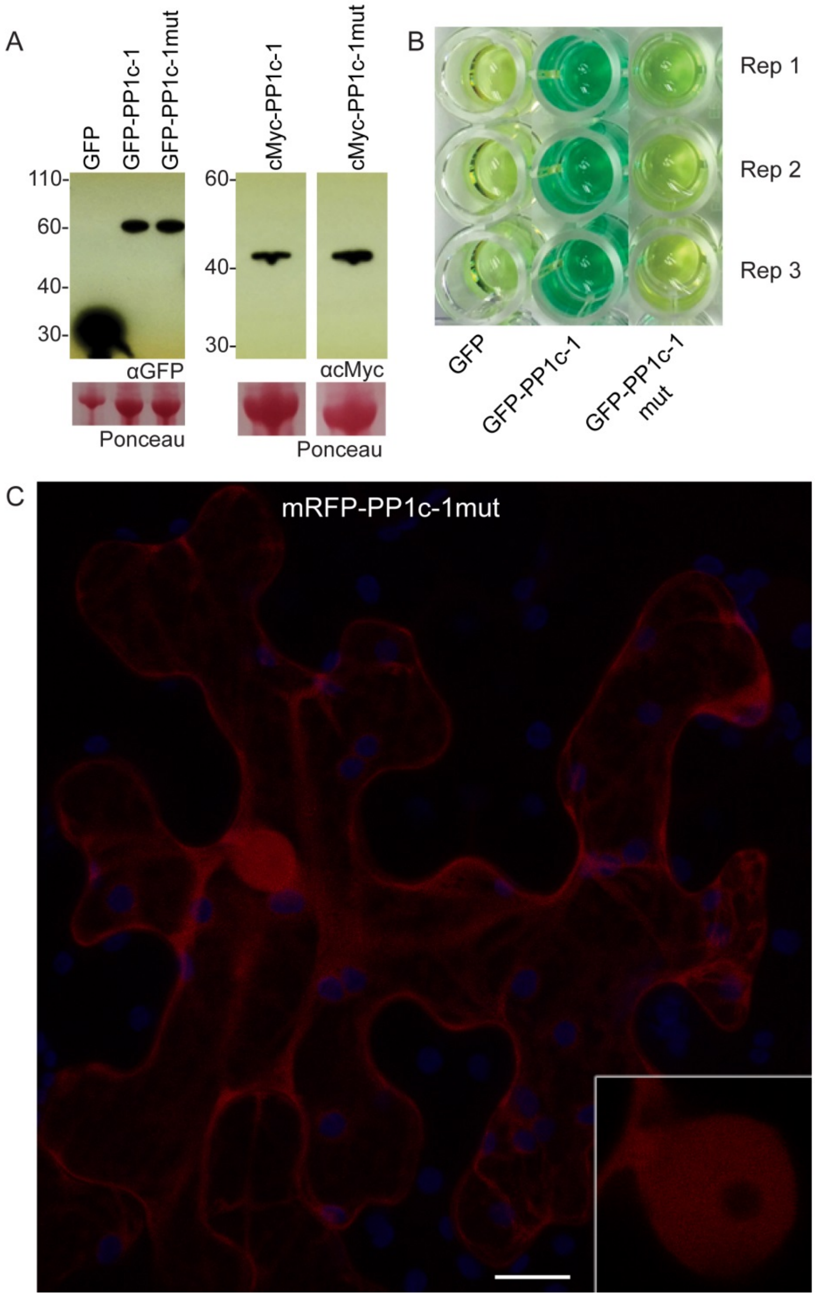

Supplementary Figure 12. PP1c-1mutant constructs are stable and do not possess phosphatase activity.

(A) Immunoblots showing the stability of GFP-PP1c-1 and GFP-PP1c-1mut used for the phosphatase activity assays. cMyc-PP1c and cMyc-PP1c-1mut used in the *in planta* virulence assays and co-immunoprecipitation are also stable. Protein size markers are indicated in kDa, and protein loading is indicated by Ponceau stain. Antibody probes are indicated (αcMyc and αGFP).

(B) Pictures show the visual readout of the phosphatase assay for the GFP empty vector (weak yellow colour), GFP-PP1c-1 (strong green colour) and GFP-PP1c-1mut (weak yellow colour).

(C) Image is a projection of a confocal z series of each of mRFP-PP1c-1mut isoform expressed alone which is evident in the cytoplasm and nucleoplasm, but absent from the nucleolus. Inset is a single optical section through one of the nuclei. Scale bar is 10 μm.

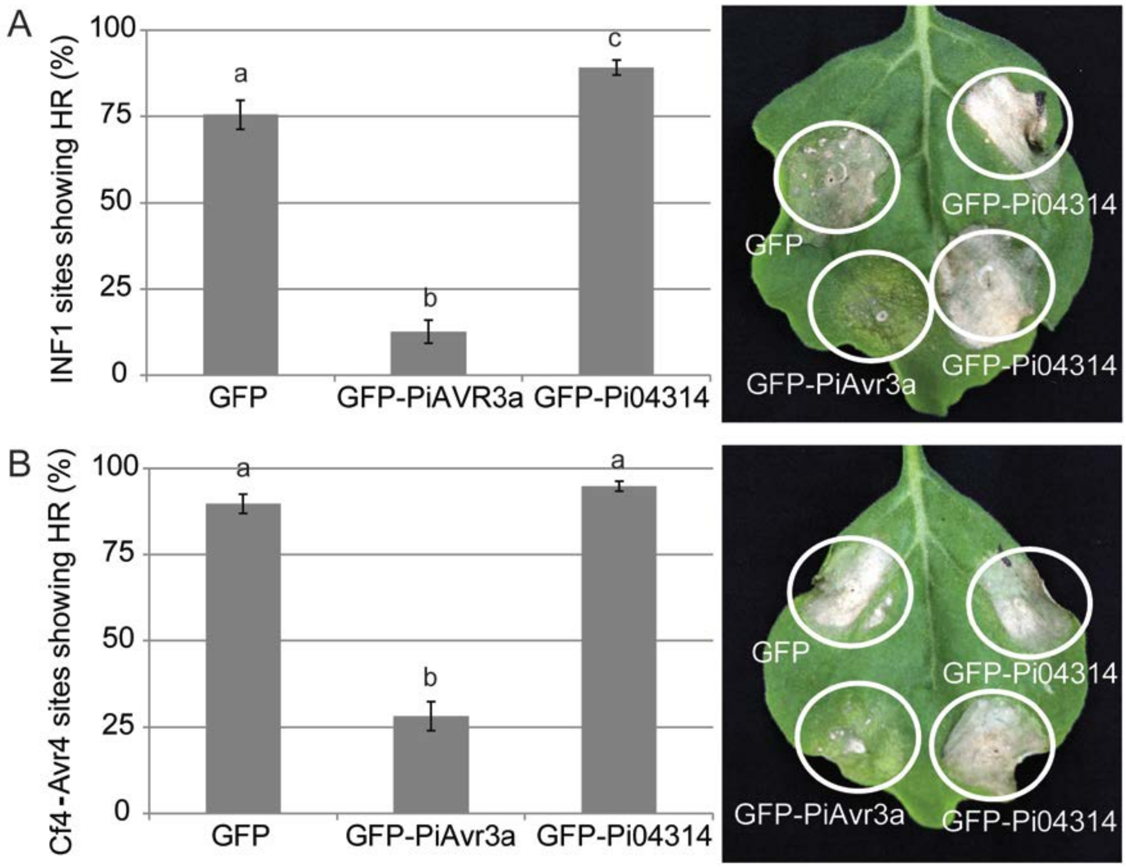

Supplementary Figure 13. Pi04314 fails to suppress INF1- or Cf4/Avr4-mediated cell death.

(A) The *P. infestans* PAMP INF1 elicited similar levels of cell death when co-expressed with either free GFP or with GFP-Pi04314, whereas cell death was significantly attenuated following co-expression with the positive control AVR3a.

(B) Co-expression of tomato Cf4 resistance with *Cladosporium fulvum* AVR4 led to a similar percentage of infiltration sites yielding cell death when co-expressed with GFP or GFP-Pi04314, compared to the positive control AVR3a. Circles indicate infiltration zones on leaves. Each graph is calculated from 3 biological replicates, each involving 18 infiltrations for each co-expression. Letters denote significant difference (ANOVA p<0.01) compared to the GFP control (given letter ‘a’).

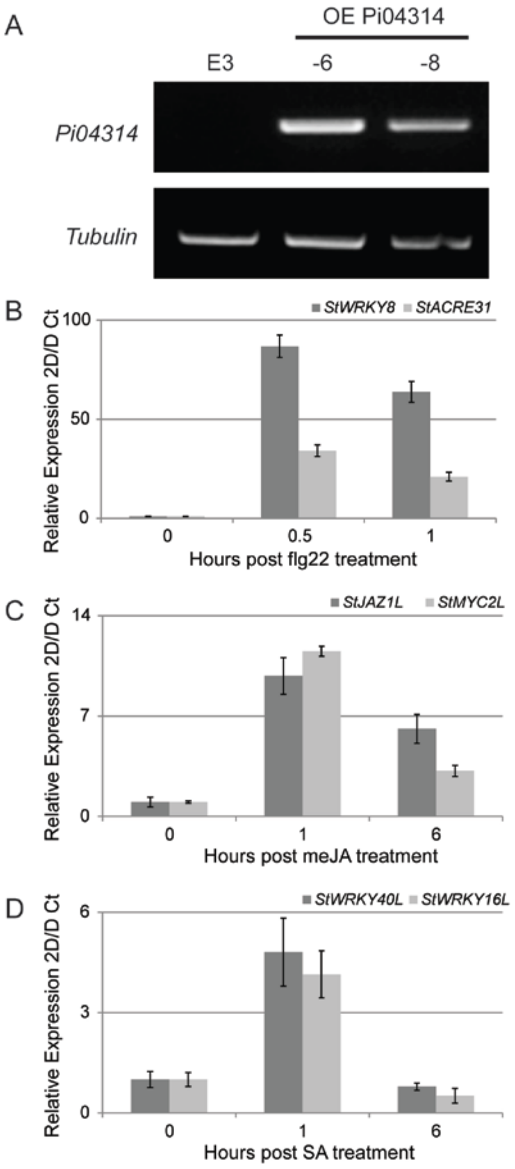

Supplementary Figure 14. Early flg22-, meJA- and SA-responsive genes for analyses of Pi04314-expressing transgenic potato lines OE-6 and OE8.

(A) RT-PCR analysis showing that *Pi04314* transcript can be detected in Pi04314-expressing potato lines OE-6 and OE-8, no such expression can be detected in the untransformed potato cv E3, whereas tubulin is detected in all cases.

(B) Relative expression (compared to untreated leaves, which were given a value of 1), using real time qRT-PCR, of *StWRKY8* and *StACRE31* in potato cv E3 after treatment with flg22.

(C) Relative expression (compared to untreated leaves, which were given a value of 1), using real time qRT-PCR, of *StJAZ1L* and *StMYC2L* in potato cv E3 after treatment with meJA.

(D) Relative expression (compared to untreated leaves, which were given a value of 1), using real time qRT-PCR, of *StWRKY40L* and *StWRKY16L* in potato cv E3 after treatment with SA. Error bars in B-D represent standard error.

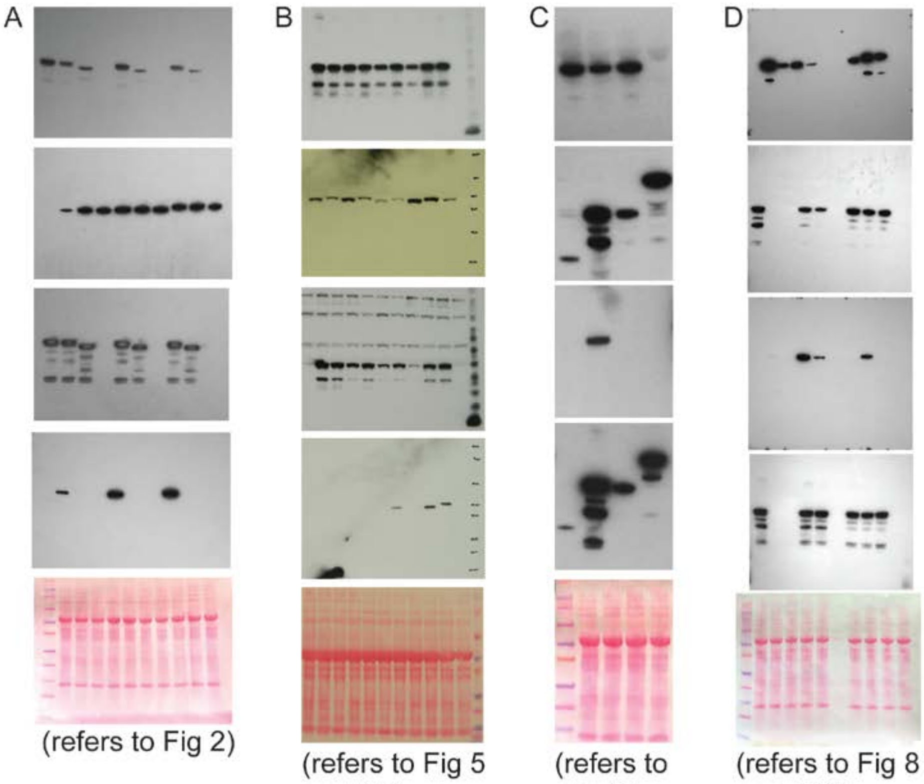

(refers to Fig 2)

(refers to Fig 5 and S9)

(refers to Fig 7)

(refers to Fig 8 and S11)

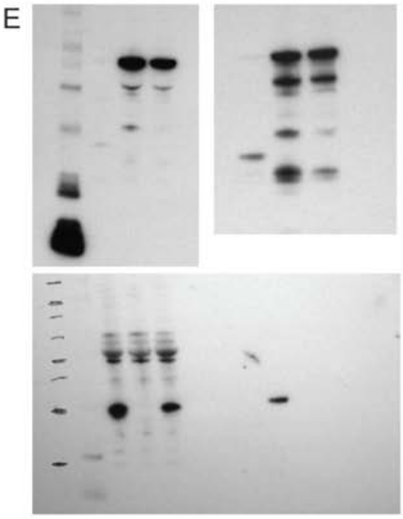

(refers to Fig 7)

Supplementary Figure 15. Original full length images for immunoblots (where those were not already used in Figures). The Figure associated with each immunoblot image is indicated below the image.

Supplementary Table 1: Primers used in this study

Primer	Use
StPP1c-1_GW_F: AAAGCAGGCTTCACCATGGCTCAAAATGGGCAGGGG	Gateway cloning primers
StPP1c-1_GW_R: GAAAGCTGGGTCTCACAAGAACCGAGGTTTTC	Gateway cloning primers
StPP1c-2_GW_F: AAAGCAGGCTTCACCATGGACCCTGCAGCTGTCGATAG	Gateway cloning primers
StPP1c-2_GW_R: GAAAGCTGGGTCTCACATCATAAACTTATT	Gateway cloning primers
StPP1c-3_GW_F: AAAGCAGGCTTCACCATGGACCAGAATGTGTTGGATG	Gateway cloning primers
StPP1c-3_GW_R: GAAAGCTGGGTCTCATGCTTTGGAATTAAA	Gateway cloning primers
StPP1c-4_GW_F: AAAGCAGGCTTCACCATGGAGGGATTGATGGAAAAAGG	Gateway cloning primers
StPP1c-4_GW_R: GAAAGCTGGGTCTCACATCCCTCCAGCCTTGG	Gateway cloning primers
Pi04314_GW_F: AAAGCAGGCTTCACCATGGTATCGACCGAAGCTAATGGG	Gateway cloning primers
Pi04314_GW_R: GAAAGCTGGGTCCTACGAGTTGGTTTTGTAGATACGAGC	Gateway cloning primers
SFI3_GW_F: AAAGCAGGCTTCACCATGGCGGTCGCTGAGACGTCA	Gateway cloning primers
SFI3_GW_R: GAAAGCTGGGTCTCAAGGTGTGGCCAGCCTC	Gateway cloning primers
NLSforwardSpe: CTAGTATGTGTACACCACCTAAGAAAAAGAAACGTAAAGTGA	NLS signal oligonucleotide
NLSreverseSpe: CTAGTCACTTTACGTTTCTTTTTCTTAGGTGGTGTACACATA	NLS signal oligonucleotide
PITG04314F: TCCATATGATGGTATCGACCGAAGCTAATGG	GM Potato cloning
PITG04314R: CGGGATCCCTACGAGTTGGTTTTGTAGATACG	GM Potato cloning
qRTNbPP1c-1F: CAATGTGAGGCTGTGGAAGA	qRT-PCR primers
qRTNbPP1c-1R: ATCGCAAAGCAAACCAGAGT	qRT-PCR primers
qRTNbPP1c-2F: CCTGTGGCAGCACTTATTGA	qRT-PCR primers
qRTNbPP1c-2R: TCGGGTATGGCAGTTGGA	qRT-PCR primers
qRTNbPP1c-3F: CCAGTGGCTGCCTTAATAGATG	qRT-PCR primers
qRTNbPP1c-3R: CCAAAGCAAATCACAGAGCAA	qRT-PCR primers
qRT_NbPP1c-4F: AAAGATTCGGATCAAATAAATGAAAT	qRT-PCR primers
qRT_NbPP1c-4R: AGGATCAGACCATAACAGATCACA	qRT-PCR primers
qRT_Nb-ef1aF:TGGACACAGGGACTTCATCA	qRT-PCR primers
qRT_Nb-ef1aR:CAAGGGTGAAAGCAAGCAAT	qRT-PCR primers
qRT_04314F: GGTTGCCCTATCTACGAGCAAA	qRT-PCR primers
qRT_04314R: GGGACCTGACGATGCTGTTTT	qRT-PCR primers
ACTAF2: CATCAAGGAGAAGCTGACGTACA	qRT-PCR primers
ACTAR2: GACGACTCGGCGGCAG	qRT-PCR primers
qRT_StPP1c-1F: CAATGTGAGGCTGTGGAAAG	qRT-PCR primers
qRT_StPP1c-1R: ACGGGAAGACAGTTGAAACAA	qRT-PCR primers
qRT_StPP1c-2F: TCTTCCTGTAGCAGCACTTATCG	qRT-PCR primers
qRT_StPP1c-2R: TCTGGGATGGCAGTTGGA	qRT-PCR primers
qRT_StPP1c-3F: CGGTCGCTGCCTTAATAGATG	qRT-PCR primers
qRT_StPP1c-3R: CCAAAGCAAATCACAGAGCAA	qRT-PCR primers
qRT_StACRE31-F: CAGGATGAATCGGATCTGAAA	qRT-PCR primers
qRT_StACRE31-R: CGGCAATCCCAATTTCTCTA	qRT-PCR primers
qRT_StWRKY8F: CCTACTGTGACATCTCATCAATCC	qRT-PCR primers
qRT_StWRKY8R: GGGTGCTCCCATTTCAGAC	qRT-PCR primers
qRT_StJAZ1LF: CTCAACAAACAGCTACCACCAC	qRT-PCR primers
qRT_StJAZ1LR: CGATGAATCACTTGATTTCTCAAT	qRT-PCR primers

qRT_StMYC2LF: AGCGTATGGAATGGAAGTGG	qRT-PCR primers
qRT_StMYC2LR: CGCAACACGTTCATCAATCT	qRT-PCR primers
qRT_StWRKY40LF: AAAATATGGTCAAAAAGTGACAAGAG	qRT-PCR primers
qRT_StWRKY40LR: CATGTTGGTGCAAATGAACAC	qRT-PCR primers
qRT_StWRKY16L_F: CAAGTGGAGGATGCAATGTG	qRT-PCR primers
qRT_StWRKY16L_R: GTTGGGAGGGAATTGTGTGT	qRT-PCR primers
qRT_StUBIF: ACACCATTGATAATGTCAAGGCTAAG	qRT-PCR primers
qRT_StUBIR: GCCATCCTCCAATTGCTTTC	qRT-PCR primers
